# Herpesvirus Genome Recognition Induced Acetylation of Nuclear IFI16 Is Essential for Its Cytoplasmic Translocation, Inflammasome and IFN-β Responses

**DOI:** 10.1371/journal.ppat.1005019

**Published:** 2015-07-02

**Authors:** Mairaj Ahmed Ansari, Sujoy Dutta, Mohanan Valiya Veettil, Dipanjan Dutta, Jawed Iqbal, Binod Kumar, Arunava Roy, Leela Chikoti, Vivek Vikram Singh, Bala Chandran

**Affiliations:** H. M. Bligh Cancer Research Laboratories, Department of Microbiology and Immunology, Chicago Medical School, Rosalind Franklin University of Medicine and Science, North Chicago, Illinois, United States of America; University of Southern California, UNITED STATES

## Abstract

The IL-1β and type I interferon-β (IFN-β) molecules are important inflammatory cytokines elicited by the eukaryotic host as innate immune responses against invading pathogens and danger signals. Recently, a predominantly nuclear gamma-interferon-inducible protein 16 (IFI16) involved in transcriptional regulation has emerged as an innate DNA sensor which induced IL-1β and IFN-β production through inflammasome and STING activation, respectively. Herpesvirus (KSHV, EBV, and HSV-1) episomal dsDNA genome recognition by IFI16 leads to IFI16-ASC-procaspase-1 inflammasome association, cytoplasmic translocation and IL-1β production. Independent of ASC, HSV-1 genome recognition results in IFI16 interaction with STING in the cytoplasm to induce interferon-β production. However, the mechanisms of IFI16-inflammasome formation, cytoplasmic redistribution and STING activation are not known. Our studies here demonstrate that recognition of herpesvirus genomes in the nucleus by IFI16 leads into its interaction with histone acetyltransferase p300 and IFI16 acetylation resulting in IFI16-ASC interaction, inflammasome assembly, increased interaction with Ran-GTPase, cytoplasmic redistribution, caspase-1 activation, IL-1β production, and interaction with STING which results in IRF-3 phosphorylation, nuclear pIRF-3 localization and interferon-β production. ASC and STING knockdowns did not affect IFI16 acetylation indicating that this modification is upstream of inflammasome-assembly and STING-activation. Vaccinia virus replicating in the cytoplasm did not induce nuclear IFI16 acetylation and cytoplasmic translocation. IFI16 physically associates with KSHV and HSV-1 genomes as revealed by proximity ligation microscopy and chromatin-immunoprecipitation studies which is not hampered by the inhibition of acetylation, thus suggesting that acetylation of IFI16 is not required for its innate sensing of nuclear viral genomes. Collectively, these studies identify the increased nuclear acetylation of IFI16 as a dynamic essential post-genome recognition event in the nucleus that is common to the IFI16-mediated innate responses of inflammasome induction and IFN-β production during herpesvirus (KSHV, EBV, HSV-1) infections.

## Author Summary

Herpesviruses establish a latent infection in the nucleus of specific cells and reactivation results in the nuclear viral dsDNA replication and infectious virus production. Host innate responses are initiated by the presence of viral genomes and their products, and nucleus associated IFI16 protein has recently emerged as an innate DNA sensor regulating inflammatory cytokines and type I interferon (IFN) production. IFI16 recognizes the herpesvirus genomes (KSHV, EBV, and HSV-1) in the nucleus resulting in the formation of the IFI16-ASC-Caspase-1 inflammasome complex and IL-1β production. HSV-1 genome recognition by IFI16 in the nucleus also leads to STING activation in the cytoplasm and IFN-β production. However, how IFI16 initiates inflammasome assembly and activates STING in the cytoplasm after nuclear recognition of viral genome are not known. We show that herpesvirus genome recognition in the nucleus by IFI16 leads to interaction with histone acetyltransferase-p300 and IFI16 acetylation which is essential for inflammasome assembly in the nucleus and cytoplasmic translocation, activation of STING in the cytoplasm and IFN-β production. These studies provide insight into a common molecular mechanism for the innate inflammasome assembly and STING activation response pathways that result in IL-1β and IFN-β production, respectively.

## Introduction

Kaposi’s sarcoma associated herpes virus (KSHV), a γ-2 herpesvirus, is etiologically associated with Kaposi’s sarcoma (KS) and primary effusion lymphoma (PEL) [1]. The hallmark of KSHV infection is the establishment of latent infection, reactivation and reinfection, and KS and PEL lesion endothelial and B cells, respectively, carry episomal KSHV latent dsDNA genome [1]. Human PEL (B) cell lines BCBL-1 and BC-3 carry >80 copies of the episomal latent KSHV genome/cell and the lytic cycle can be induced by chemicals. Purified virions from the supernatants are used for *in vitro* infection of human dermal microvascular endothelial cells (HMVEC-d) and foreskin fibroblast cells (HFF) [2].

During infection of its target cells, KSHV must be coming in contact with the host innate immune system’s pattern recognition receptors (PRR), such as Toll-like receptors (TLRs), RIG-I-like receptors (RLRs), NOD-like receptors (NLRs) and absent in melanoma 2 (AIM2)-like receptors (ALRs). TLRs on the plasma membranes and endosomes as well as the RLRs, NLRs and AIM2 in the cytoplasm recognize pathogen or danger-associated molecular patterns (PAMP/DAMP) [3, 4, 5]. KSHV infection of HMVEC-d cells induces inflammatory cytokines including the secretion of IL-1β into the supernatants which are similar to the microenvironments of KS and PEL lesions [6]. IL-1β, IL-18 and IL-33 are synthesized as inactive proforms, undergo proteolytic processing by activated caspase-1 generated by the cleavage of procaspase-1 via inflammasomes. Most of these molecular platforms are formed by homotypic interactions of a sensor protein recognizing the danger trigger, adaptor molecule ASC (apoptosis-associated speck-like protein containing CARD), and the effector procaspase-1. NLRs are cytoplasmic inflammasome sensors of foreign molecules, including ROS, K^++^, alum, bacterial products, RNA and RNA viruses replicating in the cytoplasm, while AIM2 recognizes cytoplasmic DNA including transfected DNA and DNA of pox viruses replicating in the cytoplasm [4, 7, 8, 9]. They initiate the host defenses by regulating the production of IL-1β, IL-18, IL-33 or type I interferons (IFN) α/β [7,8,9,10].

Whether innate responses recognize and respond to the presence of foreign episomal genomes of herpesviruses as well as other DNA viruses in the infected cell nuclei leading into the induction of inflammatory responses was not known initially. Our studies revealed that *in vitro* KSHV infection of endothelial cells induces caspase-1 activation via the nuclear resident gamma-interferon-inducible protein-16 (IFI16) also known as interferon-inducible myeloid differentiation transcriptional activator. Colocalization of IFI16 with viral genome in the infected endothelial cell nucleus, induction of IFI16-ASC inflammasomes by UV-inactivated KSHV and the absence of induction by lentivirus vectors expressing KSHV genes demonstrated that a) KSHV genes individually do not play a role in IFI16-inflammasome activation, b) the IFI16-inflammasome is not induced against linear integrated foreign DNA, and c) episomal KSHV genome is required for IFI16-inflammasome activation [11]. When we analyzed the gene expression in uninfected and infected HMVEC-d cells, a significant increase in caspase-1 gene expression from 2 to 24 h post-infection (p.i.), significant induction of the ASC gene only at 24 h p.i., a slight but not significant increase in IFI16 gene expression, and no increase in NLRP-1, NLRP3 and AIM2 genes were observed [11].

We have subsequently demonstrated that only the IFI16-inflammasome is constitutively induced in KSHV latently infected endothelial and PEL cells [12], as well as in B-lymphoma, epithelial and lymphoblastoid cells latently infected with γ-1 Epstein-Barr virus (EBV) [13]. Colocalization of IFI16 with the latent KSHV and EBV genome in the nuclei suggested that continuous sensing of latent genome results in the constitutive induction of IFI16-ASC inflammasomes. In addition, our studies showed that IFI16 recognizes the α-herpes simplex virus type-1 (HSV-1) genome soon after its entry into the nucleus resulting in the formation of IFI16-inflammasomes [14].

The 730 aa (1–2190 bp) IFI16 protein consists of an n-terminal ASC interacting PYRIN domain (41–261 bp), 200-amino-acid HIN I (401–895 bp) and HIN II (1043–1541 bp) domains involved in the sequence independent DNA recognition, and 2 nuclear localizing signals (NLS; 296–311 and 387–407 bp) which attribute to its nuclear entry after synthesis in the cytoplasm [15]. Though IFI16 is a predominately nuclear protein, after recognizing KSHV and HSV-1 DNA during *de novo* infection, the IFI16-ASC complex initially colocalized in the infected cell nucleus and subsequently localized in the perinuclear areas [11, 14]. Similarly, we observed the colocalization of IFI16 and ASC both in the nucleus and cytoplasm of cells latently infected with KSHV and EBV [12, 13]. Western blot analysis of *de novo* KSHV infected HMVEC-d cells showed steady levels of ASC and procaspase-1 in the nuclear fractions. Infected cells also showed higher levels of both ASC and procaspase-1 in the cytoplasmic fractions which demonstrated that ASC and procaspase-1 undergo subcellular redistribution upon infection. Active caspase-1 (p20) was detected in the nucleus of infected HMVEC-d cells at 2 and 8 h post-infection demonstrating that the inflammasome is activated upon sensing KSHV in the nucleus, and the majority of activated caspase-1 was subsequently detected in the cytoplasmic fractions at later times of infection probably to prevent caspase-1 mediated adverse activities in the nucleus. Detection of caspase-1 in the cytoplasm during *de novo* KSHV and HSV-1 infection as well as in latently infected cells demonstrated that after recognizing viral DNA in the nucleus, the newly formed IFI16-ASC inflammasome complex is transported to the cytoplasm [11, 12, 13, 14]. However, the mechanism behind the redistribution of this complex is not known.

HSV-1 infection also induced IRF-3 phosphorylation through the IFI16-STING interaction in the cytoplasm. Even though the recognition of HSV-1 genome in the nucleus via IFI16 is suggested to be the factor behind the cytoplasmic STING-IRF-3 activation and IFN-β production early during infection [16], the mechanism of post-genome detection signaling from nucleus to cytoplasm resulting in STING activation is not known. KSHV infection induces only a moderate IFN-β response early during *de novo* infection which was inhibited by a variety of early lytic and latent gene products at later times of infection [17]. The role of IFI16 in IFN-β production during KSHV infection is not known.

Using IFI16-EGFP constructs transfection in human osteosarcoma U2OS cells, Li et al., [15] studies showed that the two NLS motifs of IFI16 (aa 96–100 and aa 128–131) are essential for the entry of newly synthesized IFI16 in the cytoplasm to the normal cell nucleus. Using a FISH assay, they demonstrated that during HSV-1 (strain 17^+^) infection of U2OS cells (5 PFU/cell) containing transfected IFI16-EGFP construct, virion DNA colocalized only with full length IFI16-EGFP with intact NLS and not with mutated NLS-IFI16-EGFP that were localized in the cytoplasm. They also observed that as reported by us for KSHV [11, 12], EBV [13] and HSV-1 [14], a subset of wild type IFI16 translocated to the cytoplasm. In addition, co-IP of HSV-1 DNA-protein complexes followed by qPCR with four HSV-1 primer sets (UL30, US6,RL1 and RS1) demonstrated the nuclear IFI16 interaction with viral DNA in the nucleus. Using uninfected U2OS transfected with DNA, Li et al., [15] concluded that acetylation at the NLF motifs of IFI16 results in the cytoplasmic retention of newly synthesized IFI16 by prohibiting nuclear import, and the histone acetyltransferase p300 regulated the cytoplasmic IFI16 acetylation during transfection of DNA. However, the fate of nuclear IFI16 during HSV-1 infection, whether IFI16 undergo acetylation during HSV-1 infection, the role of p300 during viral DNA recognition in the nucleus, and the mechanism behind the IFI16 redistribution into the cytoplasm during infection was not studied [15].

Here, we demonstrate that the presence of KSHV genome in the nucleus induces the p300 mediated acetylation of IFI16 and this modification is the driving force behind the nuclear to cytoplasmic redistribution of the IFI16-inflammasome which was facilitated by Ran-GTPase. IFI16 acetylation is required for its interaction with ASC, inflammasome assembly and function. In addition, cytoplasmic redistribution of acetylated IFI16 is also essential for STING-IRF-3 mediated IFN-β production in KSHV and HSV-1 infected cells. These studies for the first time demonstrate that IFI16 acetylation is a dynamic post-herpes viral genome recognition event required for the IFI16-mediated innate responses of inflammasome induction (KSHV, EBV and HSV-1) and IFN-β production (KSHV and HSV-1).

## Results

### IFI16 recognizes the KSHV genome in the nucleus early during *de novo* infection of HMVEC-d cells leading to its redistribution to the cytoplasm

**Fig 1:**
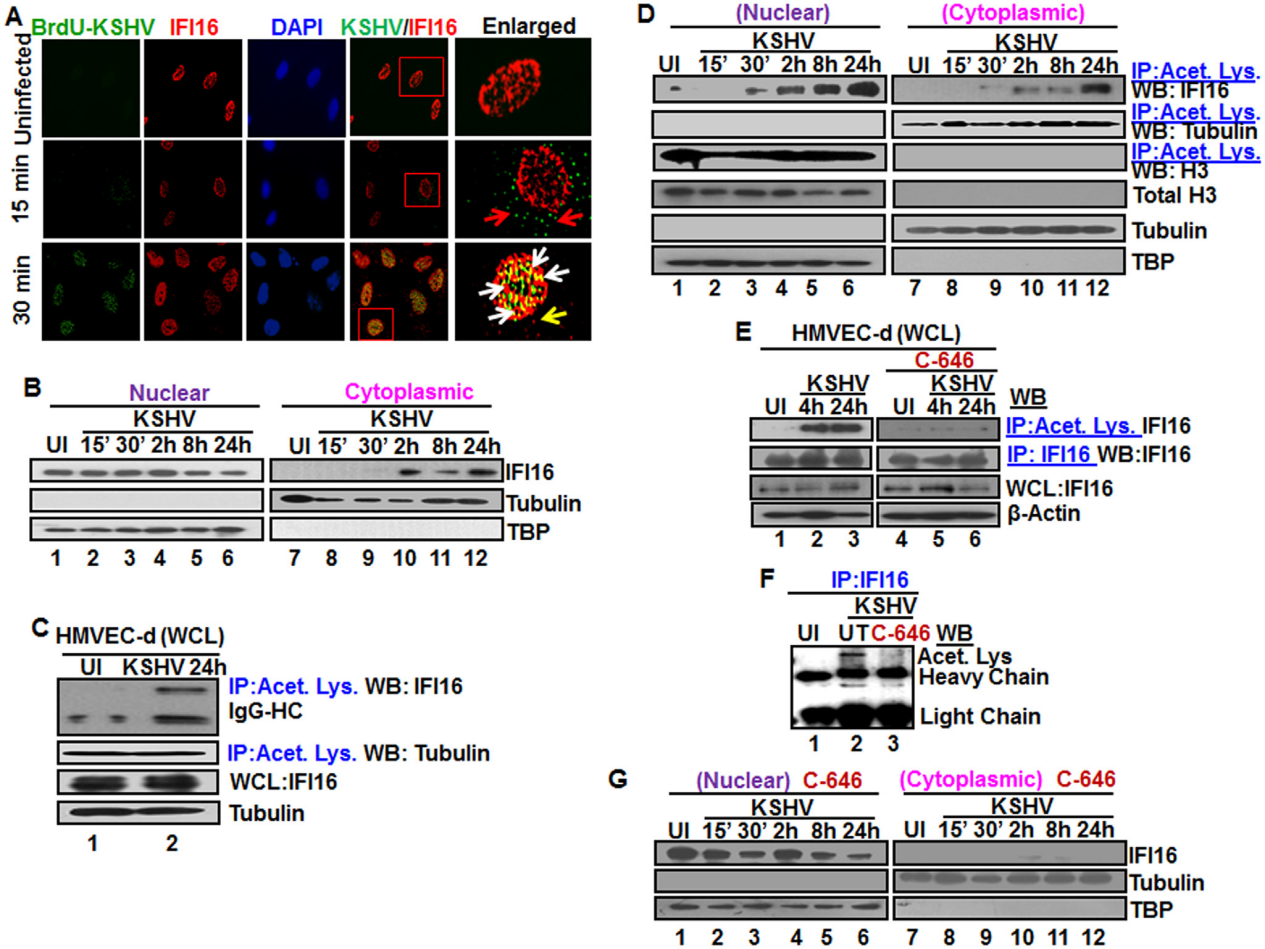
Colocalization of IFI16 with BrdU genome labeled KSHV in the nucleus, acetylation of IFI16 in the nucleus and cytoplasmic redistribution during *de novo* KSHV infection of HMVEC-d cells. **(A)** HMVEC-d cells were infected for 15 and 30 min with BrdU genome labeled KSHV (30 DNA copies/cell), processed for IFA, reacted with anti-IFI16 and anti-BrdU antibodies followed by Alexa Fluor-594/488 secondary antibodies and DAPI (blue). The boxed areas are enlarged. Red arrows: KSHV genome in the cytoplasm (green dots); yellow arrows: cytoplasmic IFI16; white arrows: colocalization of IFI16 with KSHV genome (yellow spots) in the nucleus. 60X magnification. **(B)** HMVEC-d cells were uninfected (UI) or infected with KSHV for different time points, nuclear and cytoplasmic fractions isolated and western blotted for IFI16. Tubulin and TBP were used as purity markers for cytoplasmic and nuclear fractions, respectively, and as loading controls. **(C and D)** Equal quantities of whole cell lysate (WCL) proteins from uninfected and KSHV infected (24 h) cells in NETN buffer were immunoprecipitated (IP-ed) with anti-acetylated lysine antibody and western blotted for IFI16 and tubulin (C), and cytoplasmic and nuclear proteins (D) were IP-ed with anti-acetylated lysine antibody and western blotted for IFI16, tubulin and H3. Cytoplasmic and nuclear proteins were western blotted for total H3, tubulin and TBP. **(E)** HMVEC-d cells were serum-starved without (UT = untreated) or with 1μM p300 inhibitor C-646 for 2 h, uninfected or infected with KSHV for 2 h, washed and incubated in complete medium in the presence or absence of inhibitor for 4 and 24 h. WCL in NETN buffer were IP-ed with anti-acetylated lysine or IFI16 antibodies and western blotted for IFI16. **(F)** 24 h lysates from (E) were IP-ed with anti-IFI16 and western blotted for acetylated lysine. **(G)** HMVEC-d cells treated or untreated with C-646 for 2 h were either left uninfected (UI) or infected with KSHV for various time points, nuclear and cytoplasmic fractions isolated and western blotted for IFI16.

KSHV enters HMVEC-d and HFF cells by a rapid endocytic process which is followed by the transport of genome-containing capsid to the nuclear pore vicinity, capsid disassembly and entry of the linear dsDNA into the nucleus within 15–30 min p.i., followed by the establishment of a latent infection [18]. Our studies have shown that IFI16 colocalized with the KSHV genome at 2 h p.i. in the nucleus of HMVEC-d cells [11]. To determine the earliest time of interaction of IFI16 with KSHV genome, HMVEC-d cells were infected with KSHV containing BrdU-labeled genome (BrdU-KSHV) and immunostained with anti-BrdU antibodies (Fig 1A; Table 1). IFI16 was predominantly localized in the uninfected cell nucleus (Fig 1A, top panel). By 15 min p.i., viral particles were seen in the cytoplasm and near the nuclear periphery (Fig 1A, red arrows, middle panel). In contrast, significant accumulation of viral DNA was observed at 30 min p.i. in the infected cell nuclei, and most of them colocalized with IFI16 (Fig 1A, white arrows). In addition, a few IFI16 signal spots were also detected in the cytoplasm at 30 min p.i. (Fig 1A, yellow arrow). These results suggested that IFI16 senses the KSHV genome soon after its entry into the nucleus during *de novo* infection with a concomitant redistribution to the cytoplasm.

**Table 1:** List of antibodies used in this study.

Antibody	Species	Source
IL-33	Mouse monoclonal	Santa Cruz Biotechnology, Inc., Santa Cruz, CA.
p-300	Rabbit polyclonal	Santa Cruz Biotechnology, Inc., Santa Cruz, CA.
Histone H2B	Rabbit monoclonal	Cell Signaling Technology, Beverly, MA
BrdU	Rat polyclonal	Santa Cruz Biotechnology, Inc., Santa Cruz, CA.
BrdU	Rabbit polyclonal	Rockland Inc., Gilbertsville, PA
ASC	Mouse monoclonal	MBL International, Woburn, MA
ASC/TMS1	Goat polyclonal	RayBiotech, Norcross, GA
IL-1β	Mouse monoclonal	R&D Systems, Inc., Minneapolis, MN
IFI16	Mouse monoclonal, Goat polyclonal	Santa Cruz Biotechnology, Inc
IFI16	Rabbit polyoclonal	SIGMA, St Louis, MO
H3	Rabbit polyclonal	Cell Signaling Technology, Beverly, MA
Caspase-1	Rabbit polyclonal	Invitrogen, Carlsbad, CA
Ran	Rabbit polyclonal	Abcam Inc., Cambridge, MA
Cyclin B1	Rabbit polyclonal	Cell Signaling Technology, Beverly, MA
STING	Rabbit monoclonal	Cell Signaling Technology, Beverly, MA
p-IRF-3	Rabbit monoclonal	Cell Signaling Technology, Beverly, MA
IRF-3	Mouse monoclonal	Abcam Inc., Cambridge, MA
β-actin	Mouse monoclonal	SIGMA, St Louis, MO
tubulin	Mouse monoclonal	SIGMA, St Louis, MO
TBP	Mouse monoclonal	Abcam Inc., Cambridge, MA
Alexa 594	Rabbit or Mouse	Molecular Probes, Invitrogen, Carlsbad, CA
Alexa 488	Rabbit or Mouse	Molecular Probes, Invitrogen, Carlsbad, CA
HRP tagged secondary antibody	Rabbit or Mouse	KPL Inc., Gaithersburg, MD
HRP tagged secondary antibody	Mouse (Heavy chain specific)	Alpha Diagnostics Intl. Inc. San Antonio, TX.
IRdye-680 tagged secondary antibody	Anti-mouse (IRDye 680RD)	LI COR Biotechnology, Lincoln, NE.

To determine the kinetics of IFI16 redistribution to the cytoplasm, the cytoplasmic and nuclear fractions from uninfected cells and cells infected with KSHV for various times were analyzed by western blots (WB). Consistent with the IFA results, a very faint IFI16 band was detected at 30 min p.i. in the cytoplasm which steadily increased during the observed period of 24 h p.i. (Fig 1B, lanes 9–12) with a corresponding decrease in the nuclear IFI16 levels (Fig 1B, lanes 4–6). TBP and tubulin proteins were used as markers of nuclear and cytoplasmic preparation purity and as controls for equal loading (Fig 1B, lanes 1–12). When IFA was performed to validate the biochemical data, IFI16 was predominantly in the nucleus of uninfected cells (S1A Fig, top panel). In contrast, at 30 min p.i., few IFI16 signal spots were visible in the cytoplasm which increased steadily during the observed period of 24 h p.i. (S1A Fig, red arrows). These results demonstrated that KSHV infection induces IFI16 redistribution from the nucleus to the cytoplasm as early as 30 min p.i. with steady increase thereafter.

### KSHV *de novo* infection of HMVEC-d cells induces the acetylation of IFI16 in the nucleus of infected cells

IFI16 has been shown to function as a transcriptional modulator via unknown mechanisms [19]. We theorized that acetylation of IFI16 could be one of the reasons for cytoplasmic transport since acetylation of HMGB-1 (high-mobility group protein B1) protein involved in transcription/ chromatin bending has been shown to result in HMGB-1’s translocation into the cytoplasm [20]. Furthermore, IFI16 acetylation within the NLS motifs during transfection of DNA in U20S cells promoted cytoplasmic retention by blocking nuclear import of newly synthesized IFI16 [15]. However, the fate of IFI16 during nuclear DNA sensing was not studied.

To investigate the acetylation status of IFI16 during KSHV infection, uninfected and infected cell lysates were immunoprecipitated (IP-ed) with anti-acetylated lysine antibody and western blotted for IFI16. Compared to the uninfected cells, we observed a robust increase in the acetylation of IFI16 only in the infected cells (Fig 1C, lanes 1 and 2). In contrast, equal levels of acetylated tubulin were observed in both uninfected and KSHV infected cells (Fig 1C, lanes 1 and 2). The input IFI16 and loading control tubulin were of similar levels. These results suggested that the acetylation machinery was functional in both uninfected and infected cells and KSHV infection induced increased acetylation of IFI16.

When we next investigated the kinetics of IFI16 acetylation in the nuclear and cytoplasmic fractions by co-IP experiments, as early as 30 min p.i. an appreciable level of nuclear IFI16 acetylation was observed which steadily increased during the observed 24 h p.i. (Fig 1D, lanes 2–6). Correspondingly, we detected a faint band of acetylated IFI16 in the cytoplasm at 30 min p.i., with steady increase from 2 to 24 h p.i. (Fig 1D, lanes 9–12), which corroborated the results in Fig 1B, lanes 9–12. The faint acetylated IFI16 band detected in the nucleus of uninfected cells probably represents the basal level (Fig 1D, lane 1). These detections were not due to nuclear contamination as shown by the absence of TBP and presence of tubulin in these fractions (Fig 1D). As positive control for nuclear and cytoplasmic acetylation, the proteins were IP-ed with acetylated lysine antibody and western blotted for H3 and tubulin, respectively (Fig 1D, lanes 1–12). Total H3 level was also analyzed by western blot as input control.

These results were also validated by IFA using anti-IFI16 and anti-acetylated lysine antibodies (S1B Fig). In the uninfected cells, IFI16 was detected in the nucleus and acetylated lysine signals were observed both in the nucleus and in the cytoplasm (S1B Fig, top panel). We also observed some basal level of IFI16 and acetylated lysine colocalization in the nucleus of uninfected cells (S1B Fig, UI, red arrow). In contrast, KSHV infection significantly increased the colocalization of acetylated lysine and IFI16 in the nucleus as well as in the cytoplasm in a time dependent manner (S1B Fig). Taken together, these results demonstrated that during *de novo* KSHV infection, IFI16 recognizes the viral genome with a concomitant increase in its acetylation in the nucleus and redistribution of acetylated IFI16 to the cytoplasm of the infected cells.

### Acetylation inhibitor impedes the redistribution of IFI16 from the infected cell nucleus to the cytoplasm

The cellular transcriptional coactivator protein p300 functions as a histone acetyltransferase and has been shown to be involved in the cytoplasmic acetylation of IFI16’s NLS domains [15]. To investigate the significance of nuclear acetylation of IFI16 and its redistribution, we utilized the p300 competitive inhibitor C-646. Based on the results in BCBL-1 and HMVEC-d cells incubated with various concentrations of C-646 for 4 and 24 h (S2A and S2B Fig) we selected the least toxic 1 μM concentration (5–6% cell death) for all further experiments. C-646 treatment did not interfere with viral entry or nuclear delivery of viral genome, and equal levels of the characteristic KSHV latent LANA-1 protein dots were detected in the treated and untreated cells (S2C, S2D, and S2E Fig). Significant increase in acetylation was observed in the KSHV infected cells which was reduced by C-646 treatment (S2F Fig, lanes 1–4). The specificity of C-646 was examined by the acetylation level of H2B, one of the target proteins of p300. IP with acetylated lysine antibody and WB for H2B showed six fold reduction in H2B acetylation by C-646 compared to the untreated KSHV (24 h) infected cells (S2G Fig, lanes 1 and 2). These results demonstrated that *de novo* KSHV infection induced acetylation, which is in part due to p300, can be inhibited by C-646.

To determine the effect of C-646 on IFI16 acetylation, HMVEC-d cells were either uninfected or infected with KSHV in the presence or absence of C-646, whole cell lysates IP-ed with anti-acetylated lysine antibody and western blotted for IFI16. Compared to untreated infected cells, C-646 treatment completely abolished the infection induced IFI16 acetylation (Fig 1E, lanes 1–6). Immunoprecipitation of IFI16 followed by WB for IFI16 demonstrated equal pull down; in addition, β-actin levels did not change due to treatment and showed equal loading (Fig 1E, lanes 1–6). IP of IFI16 and WB with anti-acetylation antibody also validated these results which showed decreased levels of acetylated IFI16 by C-646 treatment in infected cells (Fig 1F, lanes 1–3). To investigate the effect of C-646 on KSHV infection induced acetylation mediated cytoplasmic redistribution of IFI16, HMVEC-d cells were infected in the absence or presence of C-646, cytoplasmic and nuclear fractions isolated and western blotted for total IFI16. KSHV infection induced redistribution of IFI16 into the cytoplasm was abolished in C-646 treated cells (Fig 1G, lanes 7–12). Interestingly, we also observed that the nuclear IFI16 levels decreased at later time points by C-646 (Fig 1G, lanes 4–6) which suggested that acetylation may have a role in the stabilization of IFI16.

These results demonstrated that IFI16 acetylation during KSHV infection is dependent on p300 and acetylation is required for the redistribution of IFI16 from the nucleus to the cytoplasm after recognition of the KSHV genome in the nucleus.

### Proximity ligation assay (PLA) confirms that nuclear acetylation is required for redistribution of IFI16 to the cytoplasm

**Fig 2:**
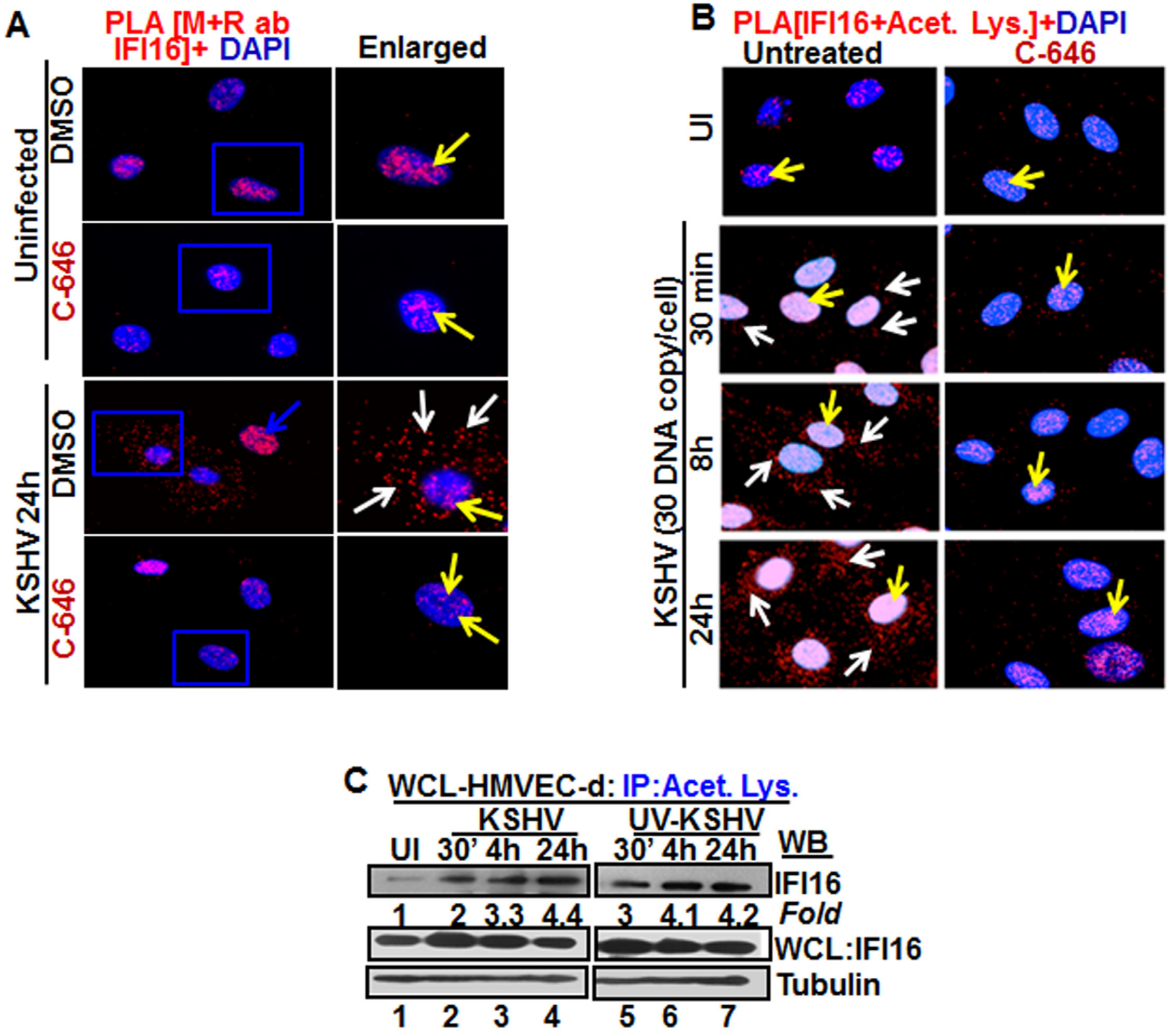
Proximity Ligation Assay (PLA) of IFI16’s acetylation and cytoplasmic redistribution during *de novo* KSHV infection of HMVEC-d cells. **(A and B)** HMVEC-d cells were preincubated with C-646 for 2 h, washed, infected with KSHV for 2 h, washed and incubated in complete medium with or without C-646. **(A)** Cells were reacted with anti-IFI16 mouse and rabbit antibodies, washed, and subjected to PLA by incubating with species specific PLA oligo probe tagged secondary antibodies, washed, incubated with ligation mixture containing ligase, amplification solution with polymerase, and fluorescently labeled oligonucleotides. The signal, detected as a fluorescent dot at 594 nm wave length, was visualized by fluorescence microscopy. Nuclei were stained with DAPI, boxed areas are enlarged and the red dots represent IFI16. The yellow and white arrows indicate the nuclear and cytoplasmic IFI16, respectively. **(B)** PLA with anti-IFI16 and anti-acetylated lysine antibodies. White arrows and yellow arrows indicate cytoplasmic and nuclear acetylated IFI16, respectively. **(C)** HMVEC-d cells uninfected or infected with live or UV-KSHV (30 DNA copies/cell) for 30 min or 2 h. The 2 h infected cells were further incubated for 4 or 24 h, and WCL proteins in NETN buffer were IP-ed with anti-acetylated lysine antibody and western blotted for IFI16. The bands were scanned and quantitated using FluorChemFC2 software and an AlphaImager system. Total IFI16 and tubulin were immunoblotted as input and loading controls, respectively.

To validate these results, we performed *in situ*-PLA which detects endogenous levels of proteins and gives the spatial distribution and localization of a single or multiple proteins (Fig 2A). PLA uses oligonucleotide-linked secondary antibodies and a fluorescence-based assay to detect closely associated proteins. If epitopes of a single protein or two protein epitopes are within 40 nm proximity, the antibody-linked oligonucleotides will ligate with adaptor oligonucleotides to form complete circles that are amplified via DNA replication and detected with fluorescent sequence-specific probes which will appear as distinct dots visible under fluorescent microscopy.

HMVEC-d cells were uninfected or infected in the presence or absence of C-646 and subjected to PLA using rabbit and mouse anti-IFI16 antibodies detecting different epitopes, and the detected red dots depict IFI16 (Fig 2A). IFI16 was predominantly nuclear in both untreated and C-646 treated uninfected cells (Fig 2A, top 2 panels, yellow arrows). In the absence of C-646, we observed abundant cytoplasmic IFI16 localization in KSHV infected cells at 24 h p.i. (Fig 2A, lower panels, white arrows), and an uninfected cell in the same field showed predominantly nuclear IFI16 (Fig 2A, blue arrow). In contrast, while IFI16 was detected in the nucleus of C-646 treated infected cells, we did not observe IFI16 redistribution in the cytoplasm (Fig 2A, lower panels). These results demonstrated that inhibition of acetylation compromised the cytoplasmic redistribution of IFI16.

To further elucidate the effect of C-646 on acetylation of IFI16, PLA was performed using anti-IFI16 and anti-acetylated lysine antibodies and the observed red dots represent the acetylated IFI16 (Fig 2B). Low levels of nuclear acetylated IFI16 PLA dots were detected both in the treated and untreated uninfected cells (Fig 2B, top panel, yellow arrows). In contrast, at 30 min p.i., acetylated IFI16 dots were appreciably increased in the nucleus with few dots visible in the cytoplasm, which increased to numerous acetylated IFI16 spots in a time dependent manner (Fig 2B, left panels, white arrows). In contrast, with C-646 treatment the acetylated IFI16 dots did not increase either in the cytoplasm or in the nucleus of infected cells (Fig 2B, lower three right panels). These studies demonstrating the reduction in cytoplasmic IFI16 redistribution by C-646 treatment validated our findings, and confirmed that IFI16 acetylation in the nucleus during KSHV infection is required for its redistribution to the cytoplasm.

### Replication incompetent KSHV *de novo* infection induces IFI16 acetylation

We have previously shown that replication incompetent UV treated KSHV (UV-KSHV) enters the cells, delivers the viral DNA into the nucleus and induces the IFI16-inflammasome [11], which demonstrated that the presence of KSHV genome is the requirement for IFI16 recognition and further consequences. When lysates from HMVEC-d cells infected with KSHV or UV-KSHV for 24 h were IP-ed with anti-acetylated lysine antibody and western blotted for IFI16, similar to live-KSHV infected cells, acetylation of IFI16 increased in a time dependent manner by infection with UV-KSHV (Fig 2C, lanes 1–7). These results suggested that the presence of viral genome is enough to induce the IFI16 acetylation process and viral gene expression is not required.

### KSHV *de novo* infection of HFF cells also induces nuclear IFI16 acetylation and redistribution in the cytoplasm

We next determined whether acetylation of IFI16 and its cytoplasmic redistribution also occur in other cell types. Compared to uninfected cells, as in HMVEC-d cells, KSHV infected HFF cells (24 h p.i.) showed increased acetylation of IFI16 which was significantly inhibited by C-646 (S3A Fig, lanes 1–4), and WB for total IFI16 showed a slight reduction in C-646 treated cells (S3A Fig, lanes 1–4). In PLA analysis, infected HFF cells in the absence of the inhibitor showed robust acetylation of IFI16 and its redistribution to the cytoplasm, which was significantly abrogated by C-646 (S3B Fig). Uninfected cells showed only a basal level of acetylated IFI16 in the nucleus (S3B Fig). Evaluation of the total IFI16 levels by PLA using mouse and rabbit anti-IFI16 antibodies revealed that IFI16 was solely nuclear in the uninfected cells (S3C Fig), while the KSHV infected cells showed IFI16 both in the nucleus and in the cytoplasm (S3C Fig). However, when the cells were treated with C-646, IFI16 was only detected in the nucleus (S3C Fig). These results demonstrated that acetylation of IFI16 is essential for its redistribution to the cytoplasm of KSHV infected HFF cells.

### IFI16 acetylation and redistribution to the cytoplasm also occur in cells latently infected with KSHV

We have shown that IFI16 recognizes the latent KSHV genome and only the IFI16-inflammasome is constitutively induced in endothelial and PEL cells carrying latent genome. Hence, we determined the acetylation status of IFI16 in these cells. Whole cell lysates from control BJAB and KSHV (+) BCBL-1 cells were IP-ed with anti-acetylated lysine antibody and western blotted for IFI16. Compared to BJAB cells, we detected increased IFI16 acetylation in BCBL-1 cells which was significantly reduced by C-646; however, total IFI16 was pulled down equally in each group (S4A Fig, lanes 1–4). Examination of total IFI16 in the cytoplasmic and nuclear fractions from untreated or C-646 treated BCBL-1 cells revealed ~6–11 fold less cytoplasmic IFI16 protein levels at 4 and 24 h of drug treatment, respectively, compared to the untreated controls (S4B Fig, lanes 4–6). These results demonstrated the acetylation dependent cytoplasmic redistribution of IFI16 in the latently infected cells. As in *de novo* infected cells, nuclear IFI16 protein levels also decreased in the presence of C-646 indicating that IFI16 stability in KSHV infected cells may be dependent upon its acetylation.

To validate these results, PLA was performed in BJAB and BCBL-1 cells using anti-IFI16 and anti-acetylated lysine antibodies (S4C Fig). Compared to the few nuclear acetylated IFI16 PLA dots in the BJAB cells (S4C Fig, upper left panel), we observed a significant increase in the acetylated IFI16 in the nucleus as well as in the cytoplasm of KSHV+ BCBL-1 cells (S4C Fig, lower left panel, yellow and white arrows, respectively). C-646 treatment resulted in a drastic reduction in acetylated IFI16 (S4C Fig, right panels). When PLA was done to examine total IFI16 and its redistribution in the absence or presence of C-646, we did not observe any cytoplasmic IFI16 in the BJAB cells (S4D Fig, upper panels). Corroborating the biochemical data in S4B Fig, increased nuclear and cytoplasmic IFI16 were observed in untreated BCBL-1 cells whereas IFI16 was mostly nuclear in the C-646 treated cells (S4D Fig, lower panels, yellow arrows). The KSHV latently infected endothelial (TIVE-LTC) and B (BJAB-KSHV) cells were also analyzed for IFI16 acetylation. IP of the whole cell lysates from control endothelial TIVE and BJAB, KSHV (+) TIVE-LTC and BJAB-KSHV cells with anti-acetylated antibody followed by IFI16 WB revealed significantly higher levels of acetylated IFI16 in both TIVE-LTC and BJAB-KSHV cells than in the KSHV negative control cells (S4E Fig, lanes 1–4). Equal amounts of IFI16 were detected in IP and in WB reactions (S4E Fig, lanes 1–4).

By PLA for IFI16 acetylation in the presence or absence of C-646, TIVE cells showed a minimal amount of acetylated IFI16 in both treated and untreated cells (S4F Fig, upper panels). In contrast, the TIVE-LTC cells showed increased levels of acetylated IFI16 both in the nucleus and in the cytoplasm (S4F Fig, lower left panel). This cytoplasmic redistribution of acetylated IFI16 was abolished by C-646 (S4F Fig, lower right panel). Total IFI16 levels in C-646 treated or untreated TIVE and TIVE-LTC cells were also analyzed by PLA using mouse and rabbit anti-IFI16 antibodies. In untreated and C-646 treated TIVE cells, IFI16 was solely nuclear (S4G Fig, upper panels). In contrast, TIVE-LTC cells showed robust IFI16 cytoplasmic redistribution (S4G Fig, lower left panel) which was significantly reduced by C-646 (S4G Fig, lower right panel).

Taken together, these results demonstrated that similar to *de novo* infected HMVEC-d cells, p300 mediated acetylation plays an important role in the cytoplasmic redistribution of IFI16 in cells latently infected with KSHV.

### IFI16 acetylation occurs during KSHV and EBV infection of primary B cells and in B cells latently infected with EBV

As an IFI16-ASC inflammasome is formed during EBV infection of B cells and in latently infected cells, we performed PLA for IFI16 and acetylated lysine in primary human B cells infected with KSHV or EBV as well as in cells latently infected with EBV (S5 Fig). Compared to uninfected cells, both KSHV and EBV infected primary B cells showed acetylation as well as cytoplasmic redistribution of acetylated IFI16 (S5A Fig). Compared to EBV negative Ramos cells, EBV latently infected Raji (latency I) and LCL (latency III) cells showed both nuclear and cytoplasmic acetylated IFI16 (S5B Fig). These results demonstrated that acetylation of IFI16 and its cytoplasmic redistribution also occur in EBV infected cells.

### Vaccinia virus infection does not induce nuclear IFI16 acetylation and cytoplasmic translocation

To determine the specificities of nuclear herpesvirus genome activation of IFI16 acetylation and its cytoplasmic distribution, we next used vaccinia virus replicating its dsDNA exclusively in the cytoplasm. The acetylation of IFI16 was not induced by vaccinia virus infection of HMVEC-d cells (S6A Fig). Only similar levels of a few dots representing basal level of acetylation were detected in the nucleus of both uninfected and vaccinia virus infected cells (S6A Fig). When mouse and rabbit antibodies were used to perform the PLA, IFI16 was predominantly detected in the nucleus of both uninfected as well as vaccinia infected HMVEC-d cells (S6B Fig). These results demonstrated that vaccinia viral DNA in the cytoplasm was not recognized by nuclear IFI16, and hence acetylation of the nuclear IFI16 and cytoplasmic translocation were not observed. These findings clearly supported our observations that the presence of nuclear KSHV, EBV and HSV-1 genomes induced the acetylation of IFI16 in the nucleus which then relocated into the cytoplasm of infected cells.

### Ran-GTPase is involved in IFI16 transport from the nucleus to the cytoplasm of KSHV infected cells

The dynamic process of exporting molecules of >50-kDa from the nucleus is initiated by exportins binding to cargo and Ran-GTP protein. The guanine-nucleotide exchange factor (GEF) of Ran that converts Ran-GDP to GTP form is in the nucleus and GTPase-activating proteins (GAPs) for Ran-GTPase are present in the cytoplasm as well as on the cytoplasmic face of the nuclear pore.

**Fig 3:**
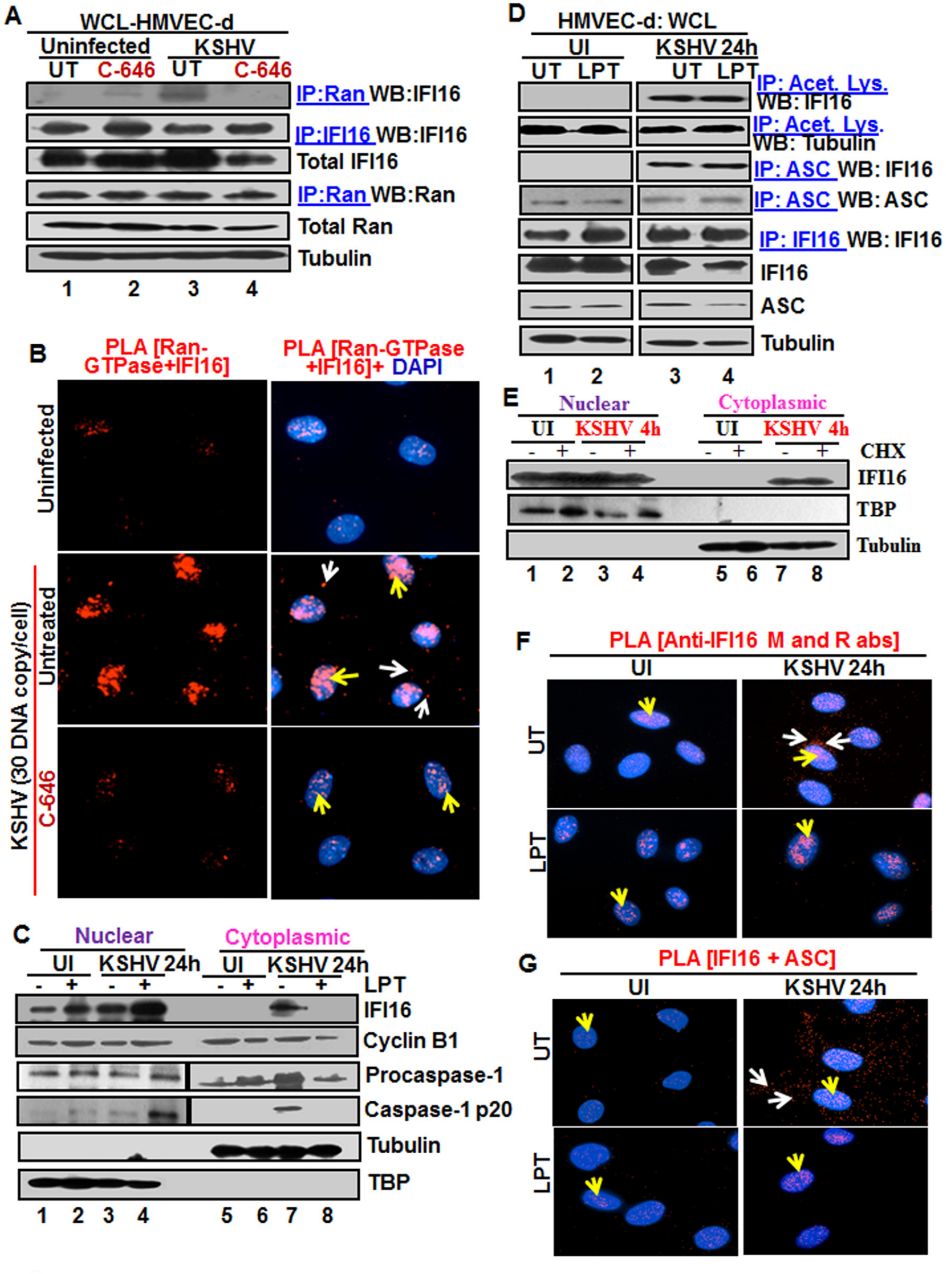
Effect of p300 inhibitor C-646 on IFI16 and Ran-GTPase association and effect of nuclear export blockage by Leptomycin B and protein synthesis inhibitor cycloheximide on the redistribution of IFI16 during *de novo* KSHV infection of HMVEC-d cells. **(A)** HMVEC-d cells preincubated with 1 μM C-646 were uninfected or infected with KSHV for 2 h, washed and incubated for 4 h without (UT) or with 1 μM C-646. WCL proteins in NETN lysis buffer were IP-ed with anti-Ran antibodies and immunoblotted for IFI16 and Ran, or IP-ed with IFI16 and WB for IFI16, samples were also immunoblotted for total IFI16, Ran and tubulin. **(B)** HMVEC-d cells preincubated with 1 μM C-646 were uninfected or infected for 2 h, washed, incubated in the absence or presence of 1 μM C-646 for 2 h, and subjected to PLA with anti-Ran-GTPase and IFI16 antibodies. White and yellow arrows denote the cytoplasmic and nuclear association of Ran and IFI16, respectively. **(C and D)** HMVEC-d cells preincubated in the presence or absence of 50 nM Leptomycin B (LPT) for 2 h were uninfected (UI) or infected with KSHV for 2 h, washed, incubated for 24 h with or without LPT. (C) Nuclear or cytoplasmic fraction proteins were western blotted for IFI16, cyclin B1, caspase-1, TBP and tubulin. (D) WCL proteins were IP-ed with anti-acetylated lysine or anti-ASC antibodies and western blotted for IFI16 and tubulin. Proteins were also IP-ed for ASC or IFI16 and WB with ASC or IFI16 antibodies, respectively. Levels of total IFI16, ASC and tubulin were detected with their respective antibodies. **(E)** HMVEC-d cells were starved for 2 h with 200 μg/ml cycloheximide (CHX), washed, infected with KSHV for 2 h, washed, incubated for 4 h with or without CHX. Cytoplasmic and nuclear fractions were subjected to western blot for IFI16, TBP and tubulin. **(F and G)** HMVEC-d cells in (C) were subjected to PLA. (F) Anti-IFI16 mouse and rabbit antibodies. Red dots: IFI16; yellow arrows: nuclear IFI16; white arrows: cytoplasmic IFI16. (G) Anti-IFI16 and anti-ASC antibodies. Red dots: IFI16-ASC association. Yellow arrows: nuclear IFI16-ASC interaction. White arrow: cytoplasmic IFI16-ASC interaction. Magnification: 60X.

To determine whether Ran is responsible for IFI16 transport from the nucleus to the cytoplasm, the lysates from uninfected or KSHV infected HMVEC-d cells (4 h p.i.) in the presence or absence of C-646 were IP-ed with anti-Ran-GTPase antibodies and WB for IFI16. Compared to the uninfected cells that showed a basal level of IFI16-RAN association (Fig 3A, lanes 1 and 2), KSHV infected cells showed robust association of IFI16 with Ran-GTPase which was inhibited by C-646 (Fig 3A, lanes 3 and 4). Comparable levels of IFI16 and Ran proteins were pulled down with their corresponding antibodies (Fig 3A, lanes 3 and 4). Higher IFI16-RanGTP association in untreated KSHV infected cells corroborated the higher cytoplasmic redistribution of IFI16 shown in the earlier figures. When PLA was performed using anti-Ran and IFI16 antibodies, consistent with the IP results, the association between these two molecules increased during KSHV infection, which was abolished by C-646 (Fig 3B). These results demonstrated that acetylation enhances the association of IFI16 with Ran-GTP during infection facilitating its transport to the cytoplasm and this association is dependent upon acetylation.

### Blocking nuclear export by Leptomycin B hampers the cytoplasmic translocation of IFI16

The nuclear resident IFI16 translocates to the nucleus after its translation in the cytoplasm via its two NLS domains and acetylation of NLS has been shown to retain IFI16 in the cytoplasm [15]. To determine whether the cytoplasmic IFI16 detected during KSHV *de novo* infection and latency represents newly synthesized IFI16 or redistributed from the nucleus, we used 50 nM Leptomycin B (LPT) to block nuclear export to the cytoplasm. This concentration of LPT was not overly toxic (6–8%) to HMVEC-d cells nor did it significantly affect the establishment of KSHV infection (S7A, S7C, and S7E Fig). When HMVEC-d cells infected with KSHV in the presence or absence of LPT were analyzed, infected cells showed enhanced cytoplasmic redistribution of IFI16 which was abolished by LPT treatment (Fig 3C, top panel, lanes 5–8). Compared to untreated cells, nuclear IFI16 increased in LPT treated cells probably due to blocked cytoplasmic redistribution (Fig 3C, top panel, lanes 1–4). Reduced cytoplasmic and increased nuclear cyclin-B1 in LPT treated cells confirmed the hampered nuclear to cytoplasmic protein transport (Fig 3C, second panel, lanes 1–8).

### Blocking nuclear export by Leptomycin B did not affect nuclear IFI16 acetylation, IFI16-inflammasome formation and function

Since IFI16-ASC-procaspase-1 assembly was initiated in the nucleus, we next examined the effect of LPT on the transport of the other components of IFI16-inflammasomes. Procaspase-1 was detected in the nucleus of untreated uninfected and infected cells (Fig 3C, third panel, lanes 1 and 3). The increased cytoplasmic procaspase-1 in untreated infected cells was significantly decreased by LPT with a corresponding increase in the nucleus (Fig 3C, third panel, lanes 7 and 8, and 3 and 4). We have previously observed the presence of cleaved caspase-1 in the nucleus of infected HMVEC-d cells at 2 h and 8 h p.i. and only in the cytoplasm at 24 h p.i. [11]. Similarly, cleaved caspase-1 was detected in the infected cell cytoplasm at 24 h p.i. which was abolished by LPT treatment with a concomitant increase in the nucleus (Fig 3C, lanes 3, 4, 7 and 8).

When cell lysates of KSHV infected HMVEC-d cells in the presence or absence of LPT were analyzed by IP with anti-acetylated antibody and IFI16 WB, IFI16 was acetylated minimally in uninfected cells and to the same extent in untreated and LPT treated infected cells; however, tubulin was acetylated in both uninfected and infected samples (Fig 3D, top 2 panel, lanes 1–4). Similarly, the IFI16 and ASC association was equal in untreated and LPT treated infected cells (Fig 3D, third panel). Equal amounts of ASC and IFI16 were pulled down with their corresponding antibodies and their total protein levels demonstrated that these proteins were available in sufficient and equal amounts in each of the experimental groups (Fig 3D, lower panels lanes 1–4).

To rule out the possibility that the detection of acetylated IFI16 is not due to the accumulation of newly synthesized IFI16 in the cytoplasm of KSHV infected cells, we blocked protein synthesis by using cycloheximide (CHX) at 200 μg/ml which was neither toxic nor affected the KSHV infection of HMVEC-d cells (S7B, S7D, and S7F Fig). Cytoplasmic and nuclear proteins from CHX treated or untreated cells left uninfected or infected with KSHV for 4 h were isolated and subjected to western blot analysis. In the presence or absence of cycloheximide, we did not detect cytoplasmic IFI16 in the uninfected cells. In contrast, we observed similar levels of cytoplasmic IFI16 in the infected cells in both the presence and absence of cyloheximide (Fig 3E, lanes 1 to 8). These results coupled with the LPT results suggested that the increased level of IFI16 in the cytoplasm of infected cells during KSHV infection is due to the translocation of acetylated IFI16 from the nucleus into the cytoplasm.

In PLA, nuclear IFI16 was detected in the untreated and treated uninfected cells (Fig 3F, left panels, yellow arrows). In contrast, in agreement with the biochemical findings, increased cytoplasmic redistribution of IFI16 in KSHV infected HMVEC-d cells was detected (Fig 3F, top right panel, white arrows) which was abrogated in LPT treated cells (Fig 3F, lower right panel). In addition, the IFI16-ASC complex was observed in both the cytoplasm and nucleus of infected cells which was constrained to the nucleus of LPT treated cells (Fig 3G, right most panels). This redistribution of IFI16-ASC complex PLA spots corroborated with earlier IFA and WB findings which demonstrated that IFI16-ASC inflammasome activation leads to redistribution of IFI16-ASC to the cytoplasm [11].

Taken together, these results demonstrated that a) blocking nuclear export by LPT did not interfere in the acetylation of IFI16, formation of IFI16-ASC complex or activation of caspase-1, b) blocking protein synthesis by CHX did not affect the cytoplasmic distribution of IFI16 from the nucleus, and c) the increased level of IFI16 in the cytoplasm in the infected cells was due to its redistribution from the nucleus and not due to newly translated cytoplasmic IFI16.

### Inhibition of IFI16 acetylation impedes formation of the IFI16-inflammasome

**Fig 4:**
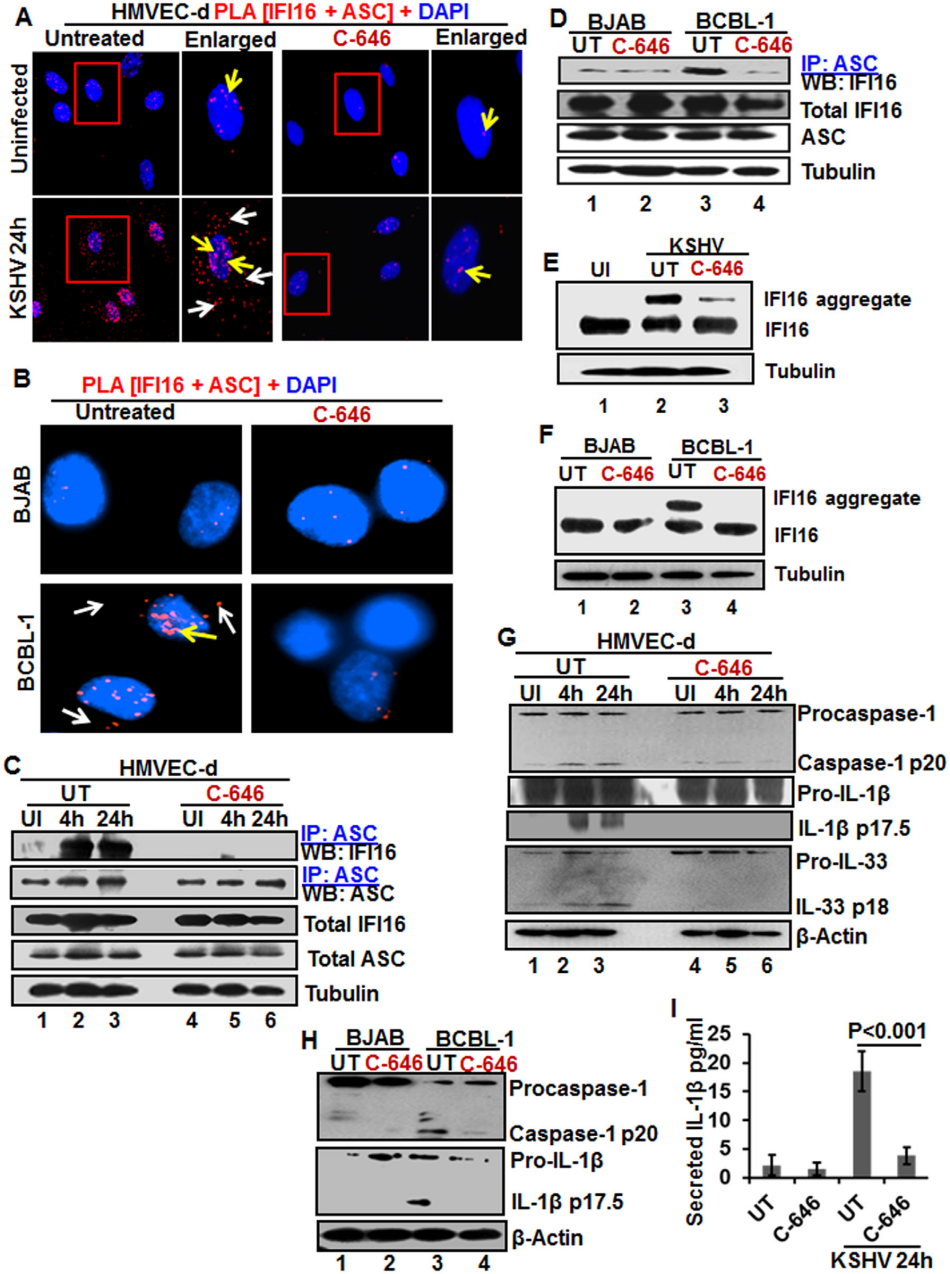
IFI16-ASC inflammasome formation, IFI16 aggregation and consequences during *de novo* KSHV infection in the presence of C-646. **(A)** HMVEC-d cells preincubated with or without 1 μM C-646 for 2 h were uninfected or infected with KSHV for 2 h, washed, incubated in complete medium for 24 h with or without C-646 and analyzed by PLA with anti-IFI16 and anti-ASC antibodies. The boxed areas are enlarged. White arrows and yellow arrows indicate cytoplasmic and nuclear IFI16+ASC complexes, respectively. **(B)** BJAB and BCBL-1 cells untreated or treated with 1 μM C-646 for 24 h were analyzed by PLA with anti-IFI16 and anti-ASC antibodies. White arrows and yellow arrows indicate cytoplasmic and nuclear IFI16+ASC complexes, respectively. **(C)** HMVEC-d cells preincubated with or without 1 μM C-646 for 2 h were washed, uninfected or infected with KSHV for 2 h, washed and incubated in complete medium with or without C-646 for 4 and 24 h. WCL proteins were IP-ed with anti-ASC antibodies and western blotted for IFI16 and ASC. Total IFI16 and ASC were used as input controls and tubulin was used as loading control. **(D)** WCL proteins from BJAB and BCBL-1 cells, untreated or treated with 1 μM C-646 for 24 h, were IP-ed with anti-ASC antibodies and western blotted for IFI16. **(E and F)** HMVEC-d cells starved with or without 1μM C-646 for 2 h, washed, infected with KSHV for 2 h, washed, incubated with complete medium for 24 h in the presence or absence of 1 μM C-646 (E). BJAB or BCBL-1 cells were untreated or treated with 1 μM C-646 for 24 h (F). Whole cell lysates were prepared in HEPES-lysis buffer, equal amount of proteins were cross-linked using glutaraldehyde and immunoblotted for IFI16. Tubulin was used as loading control. **(G)** The protein samples from panel C experiments were western blotted for caspase-1, IL-1β and IL-33. **(H)** The protein samples from panel D experiments were western blotted for caspase-1 and IL-1β. **(I)** Culture supernatants of HMVEC-d cells infected with KSHV in the presence or absence of C-646 were evaluated for IL-1β by ELISA.

Since the redistribution of acetylated IFI16 and inflammasome activation showed a similar pattern in the infected cells, we sought to determine whether the acetylation of IFI16 and IFI16-inflammasome activation are linked or independent of each other. As the association of IFI16 with the adaptor ASC is the first step in inflammasome activation, we examined these interactions by PLA. As shown in Fig 4A and 4B, in the untreated and uninfected HMVEC-d cells, few IFI16-ASC interacting PLA dots were visible in the nucleus representing the basal level of association which was reduced by C-646 treatment (Fig 4A, top panels). In contrast, in untreated KSHV infected HMVEC-d cells, we observed a robust interaction between IFI16 and ASC both in the nucleus and in the cytoplasm (Fig 4A, yellow and white arrows, respectively, lower left panel). When the C-646 treated HMVEC-d cells were infected with KSHV, the PLA dots representing IFI16-ASC interactions in the nucleus were greatly reduced with little redistribution to the cytoplasm (Fig 4A, lower right panels).

Examination of IFI16 and ASC by IFA (S8A Fig) also revealed that IFI16 was predominantly in the nucleus of the uninfected cells, while *de novo* KSHV infected HMVEC-d cells showed strong IFI16-ASC colocalization in the nucleus and redistribution to the cytoplasm (S8A Fig). When the cells were treated with C-646, only minimal IFI16-ASC interaction and cytoplasmic redistribution was detected (S8A Fig, third panel). Similarly, when BCBL-1 cells were examined by PLA and IFA, strong interactions between IFI16 and ASC were detected both in the nucleus and cytoplasm which were compromised by C-646 (Fig 4B and S8B Fig). Control BJAB cells did not show considerable IFI16 and ASC interaction in either untreated or C-646 treated cells (Fig 4B).

To confirm the IFI16-ASC interactions detected by PLA, cell lysates from uninfected and 4 and 24 h *de novo* KSHV infected HMVEC-d cells in the presence or absence of C-646 were IP-ed with ASC and western blotted for IFI16. ASC was associated with IFI16 at 4 and 24 h p.i. but no such strong association was seen in the uninfected cells, (Fig 4C, lanes 1–3). In contrast, C-646 treatment disrupted the association between IFI16 and ASC (Fig 4C, lanes 4–6). A similar amount of IFI16 was pulled down in each group either treated or not treated with C-646 (Fig 4C, lanes 1–6). A similar to primary infection, we observed increased interaction of IFI16 with ASC in the latently infected BCBL-1 cells which was greatly reduced in C-646 treated cells (Fig 4D, lanes 3 and 4). The inputs of IFI16 and ASC were similar in all groups. These results demonstrated that the presence of KSHV genome in the nucleus induced the IFI16-ASC interaction and inflammasome formation, which are dependent upon the acetylation of IFI16 in both *de novo* and latent KSHV infected cells.

The IFI16-inflammasome complex is formed by the homotypic interactions between PYD domains of IFI16 and ASC and CARD domains of ASC and procaspase-1, leading into the aggregation of IFI16 molecules [11]. To confirm that the IFI16-inflammasome complex is dependent upon the acetylation of IFI16, proteins in the cell lysates cross-linked with glutaraldehyde for 10 min were used for WB. We observed high molecular weight IFI16 aggregates in *de novo* KSHV infected HMVEC-d cells (24 h) and in BCBL-1 cells (Figs 4E, lane 2, and 6F, lane 3) and these were severely compromised by C-646 treatment (Figs 4E, lane 3, and 6F, lane 4). No such aggregation was detected in the uninfected cells (Fig 4E, lane 1, and 4F, lanes 1 and 2). These results further confirmed that acetylation of IFI16 is critical for IFI16-inflammasome formation.

### Inhibition of IFI16 acetylation impedes the function of the IFI16-inflammasome

Formation of the IFI16-ASC-procaspase-1 inflammasome leads to the generation of functional caspase-1 via auto-cleavage which results in the cleavage of the pro-forms of IL-1β, IL-18 and IL-33 cytokines. Hence, we investigated the effect of C-646 on activation of caspase-1 and its downstream cytokines production. In untreated KSHV infected HMVEC-d cells, caspase-1 activation was detected at 4 and 24 h p.i., whereas, the C-646 treated counterparts did not show considerable cleavage of caspase-1 (Fig 4G, top panel, lanes 1–6). Activation of IL-1β and IL-33 was also inhibited by C-646 treatment (Fig 4G, panels 2, 3 and 4, lanes 1–6). We also observed the inhibition of procaspase-1 and pro-IL-1β cleavages by C-646 treatment in BCBL-1 cells (Fig 4H, lanes 3 and 4), and cleavage of procaspase-1 and pro-IL-1β was not detected in BJAB cells (Fig 4H, lanes 1 and 2). The C-646 treatment did not significantly affect the viability of BJAB and BCBL-1 cells (S8C Fig). Compared to uninfected cells, increased secretion of IL-1β was observed in KSHV infected HMVEC-d culture supernatants (18.5 pg/ml) which was significantly reduced (>5-fold) by C-646 treatment (3.8 pg/ml) (Fig 4I).

We next determined the levels of active caspase-1 in BCBL-1 cells with or without C-646 by FACS using fluorescent caspase-1 detection 660-YVAD-FMK probe (S8D and S8E Fig), and the percent active caspase-1 cell populations are shown in S8F Fig. Control BJAB cells unstained or stained with FLICA-660 did not show significant caspase-1 active cells (S8D and S8F Fig). In contrast, nearly 50% of the untreated BCBL-1 cells contained active caspase-1 which was reduced to ~18–19% in C-646 treated cells (S8E and S8F Fig).

These results confirmed that acetylation of IFI16 promotes formation of functional IFI16-ASC-procaspase-1 inflammasomes leading into active caspase-1 generation and downstream cytokine production in KSHV infected cells.

### ASC is dispensable for nuclear IFI16 acetylation during *de novo* KSHV infection of HMVEC-d cells

**Fig 5:**
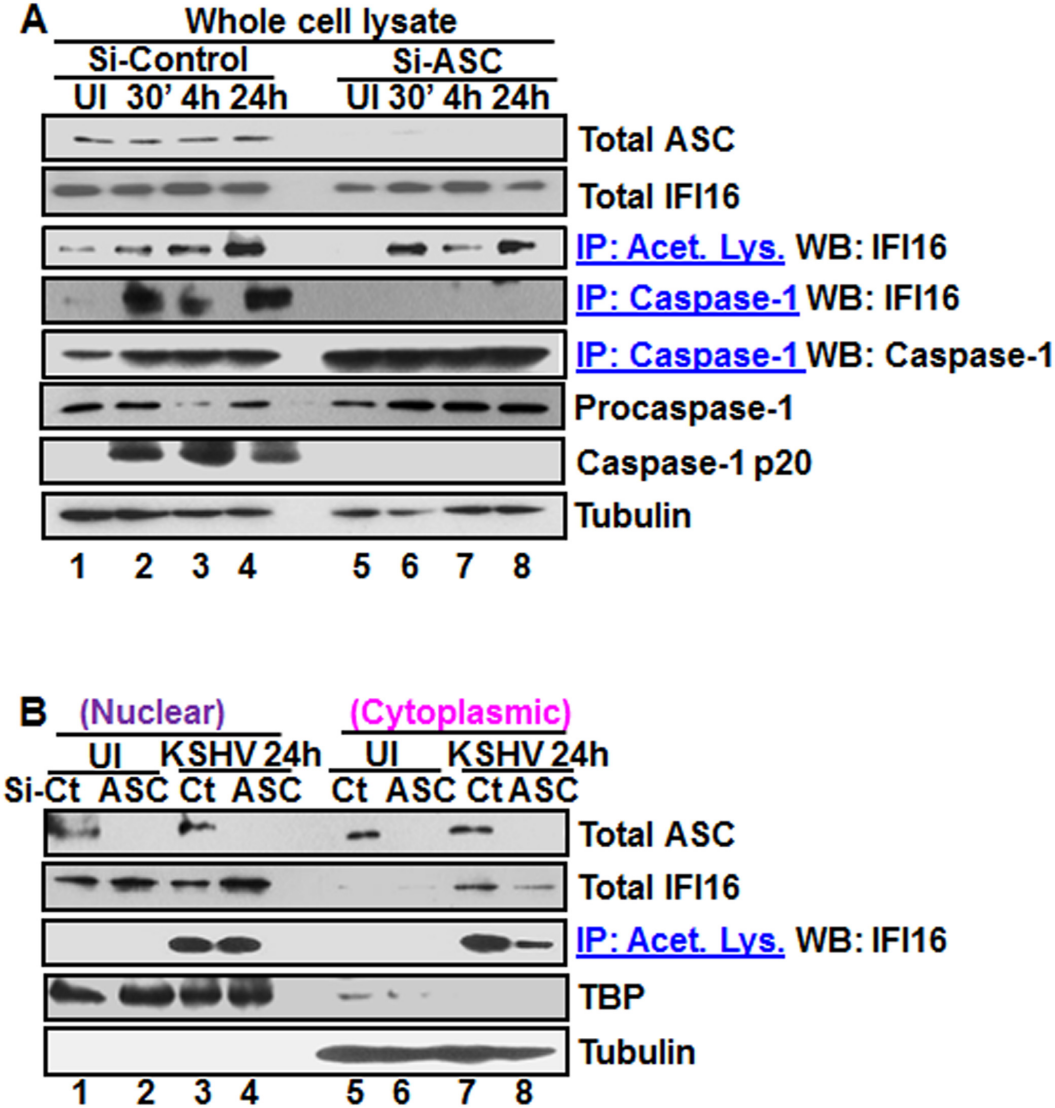
Effect of ASC knockdown on IFI16 acetylation and translocation. **(A)** HMVEC-d cells electroporated with Si-ASC and Si-control RNAs were uninfected or infected with KSHV for 30 min or 2 h, washed, the 2 h infected cells incubated for 4 and 24 h, and WCL prepared in NETN buffer. The knockdown efficiency was checked by immunoblotting for total ASC. WCL proteins were IP-ed with anti-acetylated lysine antibodies and western blotted for IFI16 or, IP-ed with anti-caspase-1 antibodies and WB for IFI16 and casapse-1. Total IFI16, caspase-1 and tubulin were tested as input and loading controls. **(B)** HMVEC-d cells uninfected or infected with KSHV for 2 h, washed, incubated with complete medium for 24 h, nuclear and cytoplasmic proteins western blotted for ASC and IFI16 or IP-ed with anti-acetylated lysine antibodies and western blotted for IFI16. TBP and tubulin were used as loading control and purity markers.

The recognition of viral genome by IFI16 leads into its increased interaction with ASC and inflammasome formation (Fig 4A–4D). Since reduction in IFI16 acetylation hampered IFI16-ASC association (Fig 4A–4D), we determined whether ASC plays roles in the acetylation of IFI16 and whether ASC associates with IFI16 after acetylation of IFI16. We knocked down the HMVEC-d cell ASC by Si-RNA electroporation and infected with KSHV. Knockdown efficiency confirmation by WB showed ~90–95% ASC reduction with no effect on IFI16 protein (Fig 5A, top two panels, lanes 1–8). The lysates from control and ASC knocked down cells were IP-ed with anti-acetylated lysine antibody and WB for IFI16. We observed the acetylation of IFI16 in both control and ASC knocked down cells (Fig 5A, third panel, lanes 1–8). As expected, in the absence of ASC formation of the IFI16-inflammasome complex was abrogated as shown by the absence of IFI16 in IP-reactions with anti-caspase-1 antibody and by the absence of caspase-1 activation in comparison to the Si-control KSHV infected cells. (Fig 5A, fourth, sixth and seventh panels, lanes 1–8). Caspase-1 was pulled down in all groups including ASC knocked down cells (Fig 5A, fifth panel, lanes 1–8). These results suggested that IFI16 acetylation occur independent of ASC.

We next determined whether IFI16 relocates to the cytoplasm in the absence of IFI16-ASC inflammasome formation. Western blot analysis of the cytoplasmic and nuclear fractions from Si-ASC uninfected and KSHV infected HMVEC-d cells showed efficient knockdown of ASC (Fig 5B, top panel, lanes 1–8). At 24 h p.i. in the Si-control cells, we observed the presence of IFI16 in the cytoplasm which was reduced by >2-fold in the ASC knockdown cells (Fig5B, second panel, lanes 7 and 8). No IFI16 was detected in the uninfected cell cytoplasm (Fig 5B, second panel, lanes 5 and 6). Interestingly, the nuclear IFI16 level was higher in KSHV infected ASC knockdown cells compared to the uninfected cells (Fig 5B, second panel, lanes 1–4). Since KSHV infection does not increase the IFI16 mRNA and protein levels [11], this moderate increase may be due to reduced, or lack of cytoplasmic redistribution of IFI16.

When the cytoplasmic and nuclear fractions were IP-ed with anti-acetylated lysine antibody and western blotted for IFI16, there was no change in the nuclear acetylated IFI16 levels in control and ASC knockdown cells (Fig 5B, third panel, lanes 3 and 4). However, similar to the total IFI16 redistribution, >3-fold reduction in the acetylated IFI16 level was observed in the cytoplasm of ASC knockdown cells (Fig 5B, third panel, lanes 7 and 8).

These results clearly demonstrated that in the absence of ASC, acetylation of IFI16 still takes place which is prior to inflammasome formation. The cytoplasmic redistribution of IFI16 in ASC knockdown cells must be inflammasome independent which might be attributed to cytoplasmic export of acetylated IFI16 either alone or in complex with other proteins. However, the reduced amount of IFI16 in the cytoplasm in comparison to Si-control suggested that the IFI16-ASC inflammasome contributes to the majority of the IFI16 detected in the cytoplasm of infected cells.

### KSHV infection induces increased interaction between IFI16 and histone acetyltransferase p300 and p300 is required for KSHV induced acetylation of IFI16

**Fig 6:**
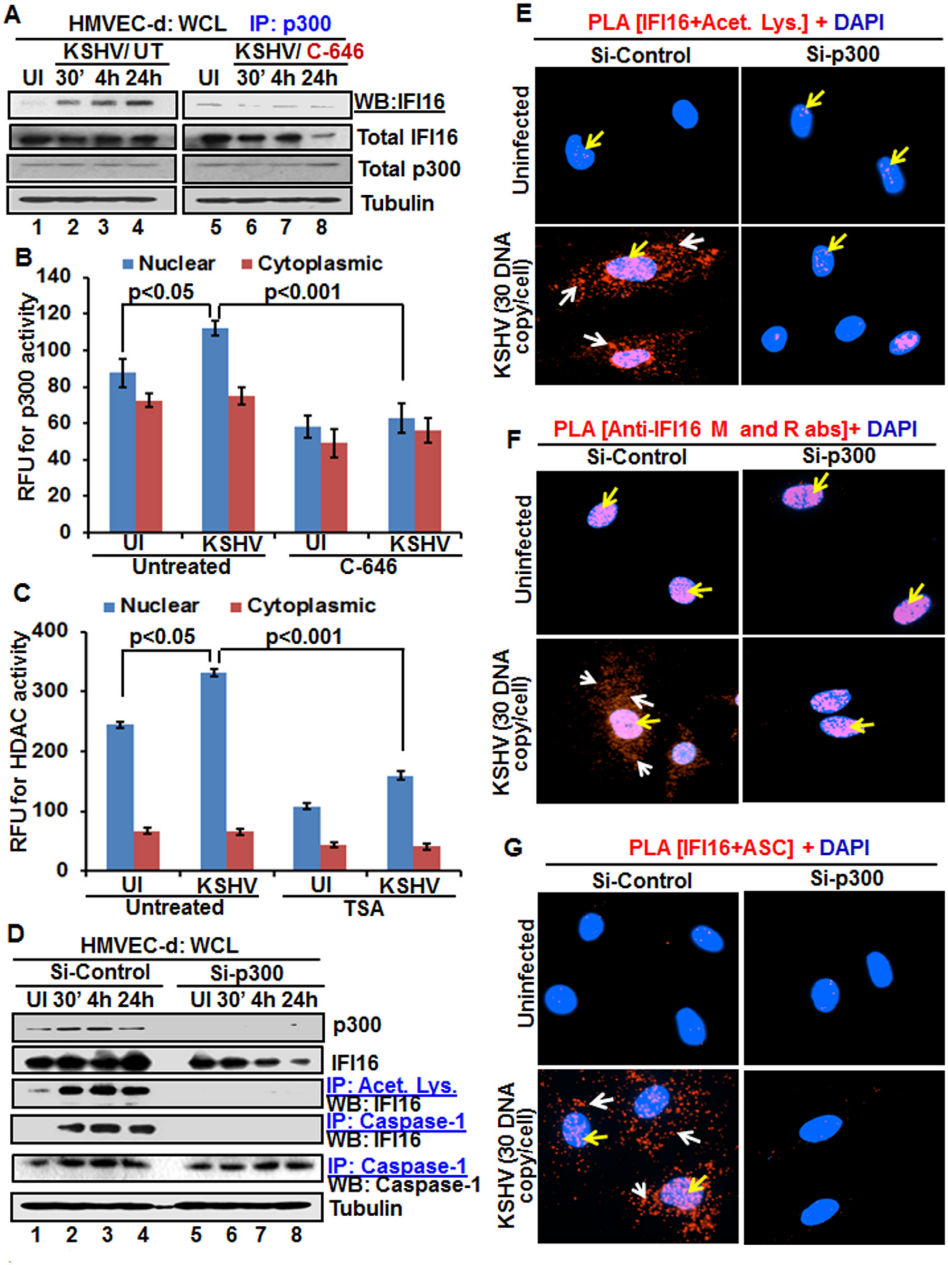
Effect of acetyltransferase p300 knockdown on IFI16 acetylation and IFI16 inflammasomes during *de novo* KSHV infection of HMVEC-d cells. **(A)** HMVEC-d cells pre-incubated with or without 1 μM C-646 for 2 h were uninfected or infected with KSHV for 30 min or 2 h, and the 2 h infected cells incubated with or without C-646 for 4 and 24 h. WCL proteins were IP-ed with anti-p300 antibodies and western blotted for IFI16. Total IFI16, p300 and tubulin were used as controls. **(B and C)** HMVEC-d cells were starved with or without p300 inhibitor C-646 or HDAC inhibitor tricostatin A (TSA), cytoplasmic and nuclear fractions evaluated for p300 (B) and HDAC (C) activities as in the Material and Methods and the results are presented here as relative fluorescence units (RFU). **(D)** The HMVEC-d cells electroporated with control or p300 Si-RNA were uninfected or infected with KSHV for 2 h, washed and incubated for various time points. WCL in NETN buffer were western blotted for total p300 and IFI16 or IP-ed with anti-acetylated lysine antibodies and western blotted for IFI16 or IP-ed with anti-caspase-1 antibodies and western blotted for IFI16 and caspase-1. **(E, F and G)** The control and p300 knockdown HMVEC-d cells were uninfected or infected with KSHV for 2 h, washed, incubated for 24 h and subjected to PLA using (E) anti-IFI16 and anti-acetylated lysine antibodies, (F) anti-IFI16 mouse and rabbit antibodies, and (G) anti-IFI16 and anti-ASC antibodies. The white and red arrows in (E) panels indicate acetylated IFI16 in the cytoplasm or nucleus, respectively. The white and red arrows in (F) panels indicate cytoplasmic and nuclear IFI16, respectively. In (G) panels, the white and red arrows indicate IFI16-ASC colocalization in the cytoplasm and nucleus, respectively.

As a follow up to C-646 inhibition of KSHV induced p300 catalyzed acetylation of IFI16, we determined the interaction of p300 with IFI16. When HMVEC-d cells infected with KSHV for 24 h in the presence or absence of C-646 were IP-ed for p300 and western blotted for IFI16, we observed increased interaction of IFI16 with p300 which was reduced to basal levels with C-646 treatment (Fig 6A, lanes 1–8).

After detection of a physical association between IFI16 and p300 during KSHV infection, we evaluated the enzymatic activity of p300 and its counterpart HDAC in the cytoplasmic and nuclear fractions of HMVEC-d cells infected with KSHV in the presence or absence of their corresponding inhibitor (1 μM C-646 for p300 and 20 μM TSA for HDAC). KSHV infection (24 h) significantly induced p300 activity in the nucleus but not in the cytoplasm of infected cells compared to uninfected cells, which was inhibited by C-646 (Fig 6B). Similarly, HDAC activity was also induced significantly in the nucleus of infected cells which was inhibited by TSA (Fig 6C). These results suggested that IFI16 acetylation is probably due to increased activity of p300. Increased nuclear p300 activation during infection further supports that acetylation of IFI16 is probably mediated by increased p300 activity in the nucleus and not in the cytoplasm. Decreased activity of enzymes by the inhibitors further verified the specificities of these assays and the functionality of C-646 and TSA (Fig 6B and 6C).

Next, we knocked down p300 to validate our inhibitor studies. Efficient p300 knockdown by Si-p300 with no effect on IFI16 and ASC protein levels was observed (Fig 6D, top three panels, lanes 1–8). The co-IP studies of anti-acetylated lysine antibodies and IFI16 demonstrated the abrogation of IFI16 acetylation in Si-p300 KSHV infected cells while Si-control infected cells showed robust IFI16 acetylation (Fig 6D, fourth panel, lanes 1–8). Similarly, the caspase-1 and IFI16 association was detected in the control group but was abrogated in p300 knockdown infected cells, and caspase-1 was pulled down in all the tested groups (Fig 6D, fourth and fifth panels, lanes 1–8). These results further validated our findings with C-646.

In PLA studies with anti-IFI16 and anti-acetylated antibodies, very few acetylated IFI16 PLA dots were observed in the nucleus of control or p300 knockdown infected cells (Fig 6E, top panels, yellow arrows). In Si-control infected cells (24 h p.i.), a high number of acetylated IFI16 dots were visible in the nucleus and in the cytoplasm (Fig 6E, lower left panel), while only a few dots, as in uninfected cells, were detectable in the p300 knockdown KSHV infected cells (Fig 6E, lower right panel). IFI16 was solely in the nucleus of uninfected cells by total IFI16 PLA (Fig 6F, top panels, yellow arrows). Similar to the acetylated IFI16, total IFI16 was found in both the nucleus and cytoplasm of Si-control infected cells, while p300 knocked down infected cells showed only nuclear IFI16 (Fig 6F, lower panels).

When PLA was performed using anti-IFI16 and anti-ASC antibodies, the red dots representing the IFI16 and ASC association were in both the nucleus and the cytoplasm of Si-control KSHV infected cells (Fig 6G, lower left panel, white and yellow arrows). In contrast, the IFI16 and ASC association was completely abrogated in p300 knockdown infected cells (Fig 6G, lower right panel).

These results further strengthened the finding that acetylation is required for the cytoplasmic redistribution of IFI16 and p300 is responsible for the acetylation of IFI16.

### Acetylation of IFI16 is required for IFN-β production in KSHV infected cells

Besides inflammasome induction in KSHV, EBV and HSV-1 infected cells, IFI16 has also been shown to be involved in the induction of IFN-β gene through its cytoplasmic activation of the STING molecule leading into phosphorylation of the transcription factor IRF-3 which subsequently translocates into the nucleus to stimulate the IFN-β gene promoter [21]. KSHV infection induces only a moderate IFN-β response early during *de novo* infection and early lytic and latent gene products inhibit this response at later times of infection [17], and the role of IFI16 in IFN-β production during KSHV infection is not defined.

**Fig 7:**
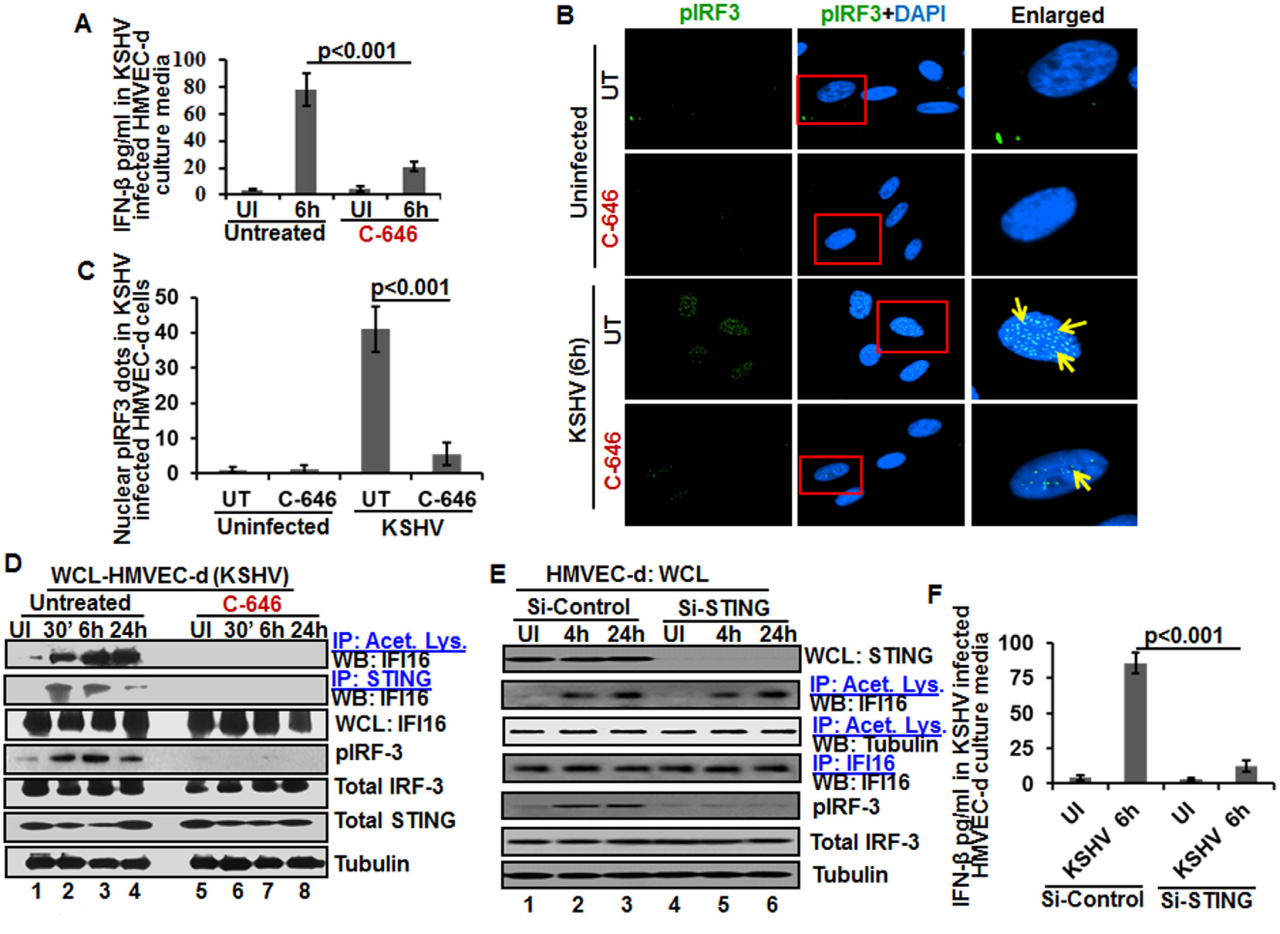
Effect of IFI16 acetylation on IFN-β production during *de novo* KSHV infection of HMVEC-d cells. HMVEC-d cells in the presence or absence of 1 μM C-646 were either left uninfected or infected with KSHV (30 DNA copies/cell) for 2 h, washed and incubated with or without 1 μM C-646 for 6 h. **(A)** Interferon-β in the culture supernatants was quantitated by ELISA. **(B)** Cells were examined by IFA with anti-pIRF-3 and Alexa Fluor- 488 secondary antibodies. Insets in the merged panels are enlarged. The yellow arrows indicate the pIRF-3 in the nucleus. **(C)** Quantitation of nuclear pIRF-3 dots. **(D)** WCL lysates in NETN buffer were IP-ed with anti-acetylated lysine and STING antibodies and immunoblotted for IFI16. Proteins were also immunoblotted for total IFI16, pIRF-3, total IRF-3, total STING, and tubulin was used as loading control. **(E)** HMVEC-d cells electroporated with control or STING Si-RNA were either left uninfected or infected with KSHV for 2 h, washed and incubated for 4 and 24 h. WCL prepared in NETN buffer were western blotted for total STING, pIRF3, total IRF3 and tubulin and IP-ed with anti-acetylated lysine antibodies and western blotted for IFI16 and tubulin or IP-ed with anti-IFI16 antibodies and western blotted for IFI16. **(F)** HMVEC-d cells electroporated with control or STING Si-RNA were uninfected or infected with KSHV for 2 h, washed and incubated for 6 h. Cells culture supernatants were used to measure the levels of IFN-β by ELISA.

When we analyzed the role of IFI16 and its acetylation in IFN-β production, we detected IFN-β in the supernatants of KSHV infected HMVEC-d cells at 6 h p.i., which was significantly reduced by >4 fold by C-646 treatment (Fig 7A). A significant level of phosphorylated IRF-3 detected in the nucleus at 6 h p.i. was reduced in C-646 treated cells (Fig 7B and 7C). Immunoprecipitation with anti-acetylated lysine antibody followed by IFI16 WB revealed the presence of acetylated IFI16 from 30 min to 24 h p.i. in KSHV infected cells, which was abolished by C-646 treatment (Fig 7D, top panel, lanes 1–8). IP reactions with anti-STING antibodies demonstrated the increased IFI16-STING interaction from 30 min to 6 h p.i. and its decrease at 24 h p.i., which was abolished by C-646 (Fig 7D, second panel, lanes 1–8). Similarly, the levels of pIRF-3 increased in untreated KSHV infected cells which were abolished in C-646 treated infected cells (Fig 7D, fourth panel, lanes 1–6).

### IFI16 acetylation in *de novo* KSHV infected cells is upstream to STING activation

Next, we knocked down STING in HMVEC-d cells to determine whether IFI16 acetylation is upstream or downstream to STING activation. Efficient knockdown was achieved by electroporation using STING specific Si-RNA (Fig 7E, top panel). KSHV infection was not affected under these conditions as shown by the increased IFI16 acetylation which was not affected by STING knockdown (Fig 7E, second panel) which suggested that IFI16 acetylation is upstream to STING activation. Control tubulin protein was acetylated in uninfected and infected cells, and an equal amount of IFI16 was pulled down in all groups (Fig 7E, fourth panel). IRF-3 was phosphorylated post-KSHV infection which was hampered in STING knockdown cells; however, total IRF-3 was detected in equal amounts in all the groups and results with tubulin showed equal loading (Fig 7E, last three panels). An increased level of IFN-β was observed in the supernatants of HMVEC-d cells infected with KSHV which was significantly reduced by STING knockdown (Fig 7F).

These studies demonstrated that acetylation during KSHV infection induced IFI16 acetylation is required for its cytoplasmic interaction with STING, pIRF-3 induction, and IFN-β production, IFI16 acetylation is upstream to STING activation and STING does not play any role in IFI16 acetylation.

### Acetylation of IFI16 is required for IFN-β production in HSV-1 infected cells

**Fig 8:**
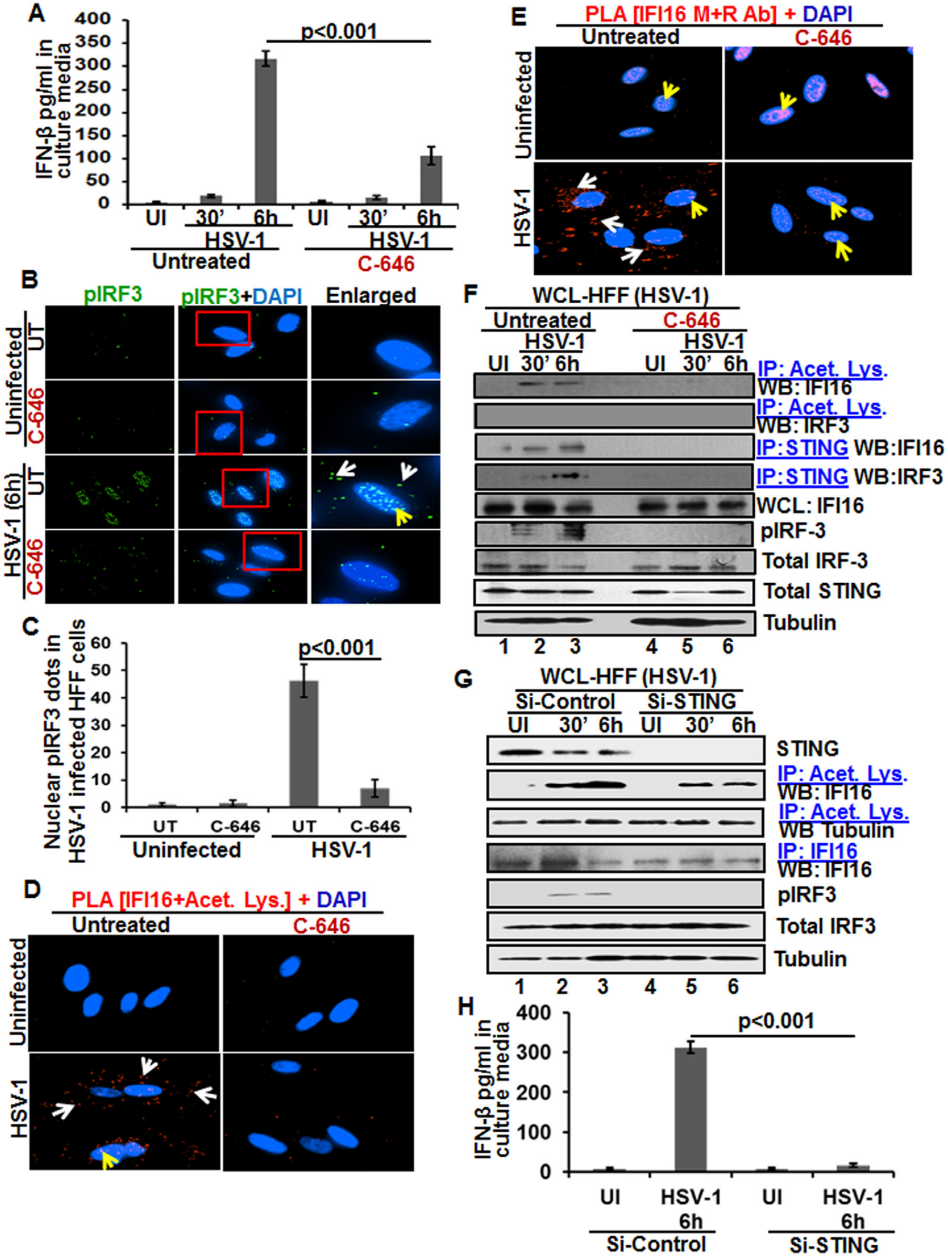
Effect of IFI16 acetylation on IFN-β production during *de novo* HSV-1 infection in HFF cells The HFF cells uninfected or infected with HSV-1 (1 PFU/cell MOI) in the presence or absence of 1 μM C-646 for 30 min or 2 h were washed and incubated with or without 1 μM C-646 for 6 h. **(A)** IFN-β in the culture supernatants was quantitated by ELISA. **(B and C)** Cells were examined by IFA with anti-pIRF-3 and Alexa Fluor-488 secondary antibodies. Insets in the merged panels are enlarged. The white and yellow arrows indicate the pIRF-3 in the cytoplasm or nucleus, respectively. Quantitation of nuclear pIRF-3 dots is shown in (C). **(D)** PLA with anti-IFI16 and anti-acetylated lysine antibodies. White and yellow arrows indicate the cytoplasmic and nuclear acetylated IFI16, respectively. **(E)** PLA with mouse and rabbit anti-IFI16 antibodies. White and yellow arrows indicate the cytoplasmic and nuclear IFI16, respectively. **(F)** WCL lysates in NETN buffer were IP-ed with anti-acetylated lysine and STING antibodies and immunoblotted for IFI16 and IRF-3. Proteins were also immunoblotted for total IFI16, pIRF-3, total IRF-3, and total STING with tubulin used as loading control. **(G)** HFF cells were transfected with control or STING Si-RNA with electroporation, either left uninfected or infected with HSV-1 (1 PFU/cell MOI) for 30 minutes or 2 h, washed and incubated for 6 h. WCL prepared in NETN buffer were western blotted for total STING, pIRF3, IRF3 and tubulin or IP-ed with anti-acetylated lysine antibodies and western blotted for IFI16 and tubulin, or IP-ed with anti-IFI16 antibodies and western blotted for IFI16. **(H)** HFF cells electroporated with control or STING Si-RNA were uninfected or infected with HSV-1 for 2 h, washed and incubated for 6h. Cell culture supernatants were used to measure the levels of IFN-β by ELISA.

We and others have shown that HSV-1 infection of HFF cells also induced the IFN-β gene and secretion of IFN-β which was dependent upon IFI16 and IRF-3 [16,22]. We utilized C-646 to determine whether IFI16 acetylation has any role in IFN-β production during HSV-1 infection. C-646 did not show any cytotoxic effects on HFF cells nor did it affect the infectivity of HSV-1 (S9A and S9B Fig). At 30 min post HSV-1 infection, 20 and 16 pg/ml of IFN-β was detected in untreated and C-646 treated supernatants, respectively (Fig 8A). At 6 h p.i., 317±16.5 pg/ml of IFN-β was detected in untreated cells whereas significant (>67%; p<0.001) inhibition of IFN-β production was observed in the C-646 treated cells (107±19.4 pg/ml; Fig 8A).

When we examined the phosphorylation of IRF-3 by IFA, compared to the uninfected cells, at 6 h p.i., appreciable levels of phosphorylated IRF-3 were detected in the nucleus and in the cytoplasm (Fig 8B, third panel). In contrast, C-646 treatment prior to infection reduced these levels especially in the nucleus (Fig 8B, fourth panel, and 8C).

In PLA studies, similar to KSHV infected HMVEC-d cells, we observed the cytoplasmic redistribution of acetylated IFI16 in HSV-1 infected HFF cells (6 h p.i.) which was inhibited by C-646 (Fig 8D). IFI16, which was predominantly nuclear in the uninfected HFF cells, was detected in the cytoplasm of HSV-1 infected cells which was abrogated by C-646 (Fig 8E). The observed reduction in the total as well as acetylated IFI16 levels is probably due to the degradation of IFI16 by HSV-1 via its ICPO protein [14].

When the whole cell lysates in the presence or absence of C-646 were IP-ed with anti-acetylated lysine antibody and WB for IFI16 and IRF-3, acetylation of IFI16 was observed as early as 30 min p.i., which was abolished by C-646 (Fig 8F, top panel, lanes 1–6). Acetylation of IRF-3 was not observed (Fig 8F, second panel). In IP-reactions with anti-STING antibodies, increased levels of IFI16 and IRF-3 were detected at 30 min and 6 h p.i. which demonstrated that IFI16 interacts with STING and STING interacts with IRF-3 (Fig 8F, third and fourth panels). These interactions were abrogated by C-646 (Fig 8F, third and fourth panels, lanes 4–6). The level of pIRF-3 increased in untreated HSV-1 infected cells, whereas it was absent in C-646 treated infected cells (Fig 8F, sixth panel, lanes 1–6). As expected, IFI16 levels decreased at 6 h p.i., and in contrast, the IFI16 level was unchanged with C-646 which further suggested that acetylation might be facilitating the stability of IFI16.

Similar to KSHV infected cells, IFI16 acetylation was not affected by STING knockdown during HSV-1 infection (Fig 8G, lanes 1–6). Equal levels of IFI16 was pulled down in both Si-control and in STING knockdown HSV-1 infected cells, and IRF-3 was activated in Si-control HSV-1 infected cells and not in STING knockdown cells (Fig 8G, lanes 1–6). HSV-1 infection induced IFN-β production was hampered in STING knockdown cells (Fig 8H).

Together, these results demonstrated that as in KSHV infected cells, IFI16 acetylation and its translocation to the cytoplasm in HSV-1 infected cells is also critical for its interaction with STING in the cytoplasm, subsequent STING interaction with IRF-3, phosphorylation of IRF-3, and nuclear translocation of pIRF-3 leading into IFN-β production.

### Acetylation is not obligatory for IFI16’s ability to recognize nuclear viral genomes

**Fig 9:**
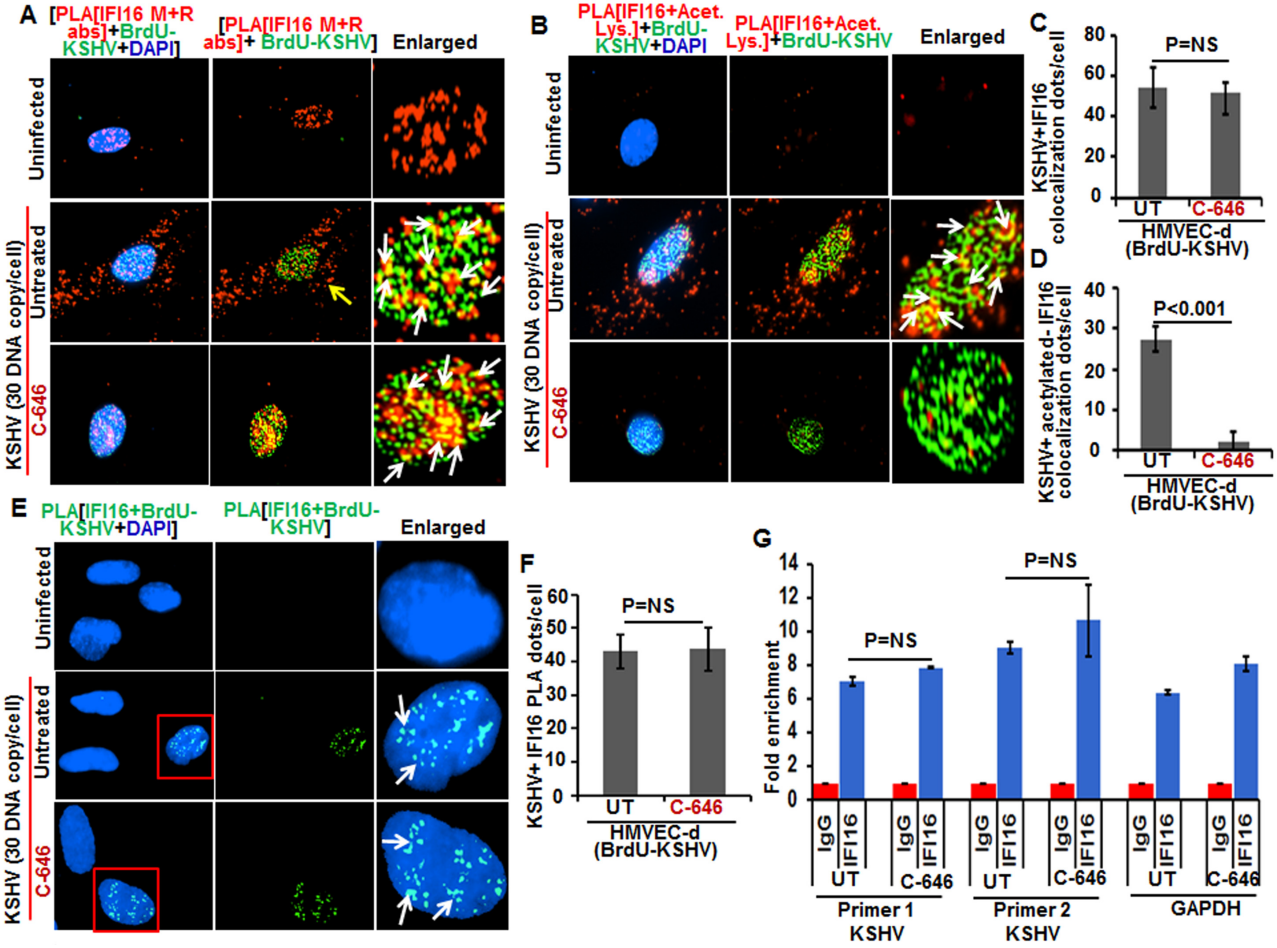
Effect of acetylation inhibition by C-646 on the ability of IFI16 to recognize and bind the KSHV genome. **(A and B)** HMVEC-d cells pre-incubated with or without 1 μM C-646 for 2 h were washed, infected with BrdU genome labeled KSHV (30 DNA copies/cell) for 2 h, washed and incubated with or without 1 μM C-646 for 4 h. Cells were processed, incubated with anti-BrdU antibodies, washed, and reacted with Alexa Fluor-488 (green) secondary antibodies to detect BrdU-KSHV. These slides were subjected to PLA **(A)** with mouse anti-IFI16 and rabbit-IFI16 antibodies or **(B)** with anti-IFI16 and anti-acetylated lysine antibodies. Boxed areas are enlarged. The yellow arrows in (A) and (B) indicate the cytoplasmic IFI16 or acetylated IFI16, respectively. The white arrows in (A) indicate colocalization of IFI16 PLA spots with BrdU labeled KSHV genome. The white arrows in (B) indicate colocalization of acetylated IFI16 PLA spots with BrdU labeled KSHV genome. **(C and D)** Bar graph of quantitation of colocalization of IFI16 or acetylated IFI16 PLA spots with BrdU labeled KSHV genome in (A) and (B) panels. At least ten fields with a minimum of 3–4 cells/field were counted. **(E)** The above described cells were subjected to PLA using anti-BrdU and anti-IFI16 antibodies to detect the direct association of IFI16 with BrdU-labeled KSHV genome. Boxed areas are enlarged. The PLA dots indicated by white arrows represent the association between IFI16 and BrdU-labeled KSHV genome. **(F)** PLA spots from 10 fields with at least 3–4 cells/field in (E) were quantitated and presented as a bar graph **(G)** Chromatin immunoprecipitation was performed using anti-IFI16 antibody as described in the Material and Methods. Untreated BCBL-1 cells (3 x 10^7^) or cells treated with 1 μM C-646 for 24 h were cross-linked with formaldehyde and sonicated to obtain DNA fragments between 200–400 bps. q-PCR was performed with primers for two different regions on the KSHV genome and one control for GAPDH, and the results are presented as fold enrichment of immunoprecipitated DNA. The p values were calculated using Student’s T test. NS (non-significant).

Since recognition of KSHV, HSV-1 and EBV genome by IFI16 in the nucleus of infected cells leads to inflammasome activation [11,13,14], we determined whether acetylation of IFI16 is required for its ability to sense the viral genome. Cells were infected with BrdU-KSHV for 6 h in the presence or absence of C-646, IFA was performed for BrdU followed by PLA using anti-IFI16 mouse and rabbit antibodies (Fig 9A). IFI16 was mostly nuclear in the uninfected cells. At 6 h p.i., we observed the appreciable colocalization of IFI16 with KSHV genome in the nucleus of both untreated as well as C-646 treated HMVEC-d cells (Fig 9A, enlarged panels). As before, we observed IFI16 redistribution in the cytoplasm which was absent in C-646 treated cells (Fig 9A). Increased associations of acetylated IFI16 with BrdU-KSHV were observed in untreated cells (Fig 9B, enlarged panels, and white arrows) which were completely abrogated by C-646 (Fig 9B, lower enlarged panel, and 9D). The IFI16-KSHV genome colocalization spots in untreated and C-646 treated cells were similar and the difference was not statistically significant (Fig 9C). Interestingly, the levels of acetylated IFI16 molecules associated with BrdU-KSHV were about 50% less than that of the total IFI16 associated with viral genome (Fig 9C and 9D).

To confirm the direct association of IFI16 with KSHV genome, we performed PLA and chromatin immunoprecipitation (ChIP) assays. To detect the direct binding of IFI16 with KSHV genome, we infected HMVEC-d cells with KSHV with BrdU labeled genome and performed the PLA using anti-BrdU and anti-IFI16 antibodies as this will give signal only when KSHV genome and IFI16 interact and are at close proximity (<40 nm). In the PLA reactions, we observed that the number of IFI16-KSHV genome colocalization spots were similar in both the untreated as well as C-646 treated KSHV infected cells (Fig 9E, white arrows, and 9F). These results further corroborated the Fig 9A results and demonstrated that IFI16 acetylation does not play any role in viral genome recognition. Similar results were also observed in HFF cells infected with BrdU genome labeled HSV-1 (S9C and S9D Fig).

We carried out the ChIP assay of KSHV infected BCBL-1 cells with and without C-646 treatment by pulling down the DNA associated with IFI16 and performed qPCR using primers for two different locations of KSHV and with a control GAPDH primer (Fig 9G). We did not observe any significant changes in the binding of IFI16 with KSHV genome by C-646 treatment (Fig 9G).

These results suggested that a) IFI16 directly associates with KSHV and HSV-1 genomes, b) the acetylation of IFI16 is not required for genome recognition, c) IFI16 acetylation occurs as a dynamic post-genome recognition event, and d) post-acetylation, IFI16 probably moves away from the genome for the formation of its complexes and eventually leading to its cytoplasmic translocation.

## Discussion

IFI16, a member of the ALR family, has emerged as a critical sensor against both nuclear and cytoplasmic DNA with pivotal roles in inflammasome activation and IFN production [11, 21]. However, how the inflammasome formed as a consequence of recognition of herpesviral genomes in the nucleus by IFI16, followed by cytoplasmic accumulation of the IFI16-ASC complex, and how HSV-1 and KSHV genome recognition in the nucleus via IFI16 lead to STING-IRF-3 activation in the cytoplasm and subsequent IFN-β production were not known. Our comprehensive studies for the first time demonstrate that acetylation of IFI16 after recognizing the viral genome occurs as a dynamic post-genome recognition event that is common to the IFI16-mediated innate responses of inflammasome induction and IFN-β production during herpesvirus infections.

**Fig 10:**
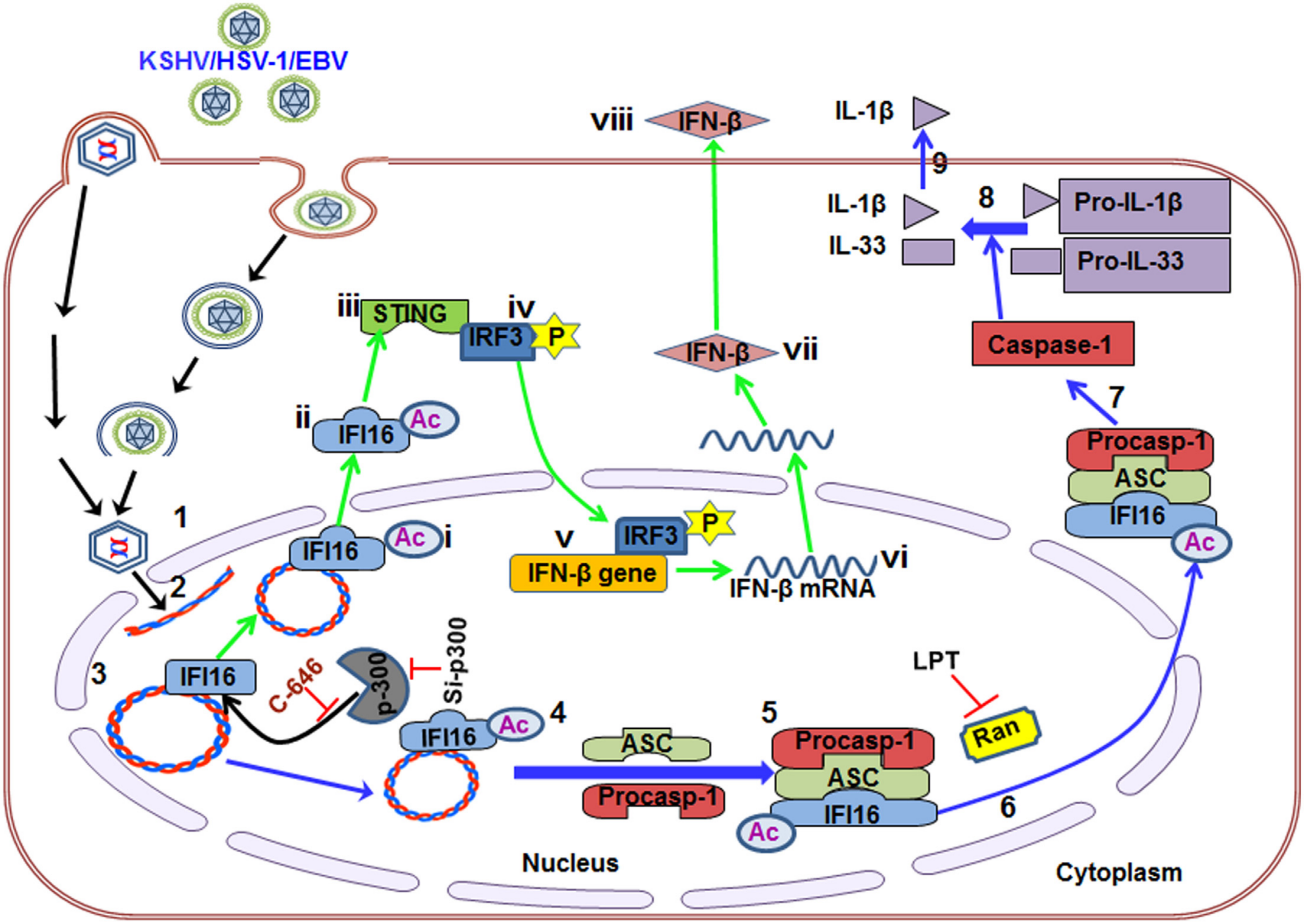
Schematic model depicting herpesvirus infection induced IFI16 acetylation and its role in inflammasome and IFN-β production. Results presented here demonstrate viral DNA entry into the nucleus (1–2) leads to recognition through IFI16 (3) followed by IFI16 acetylation (4). The acetylated IFI16 forms an inflammasome complex (5) which is translocated to the cytoplasm via RanGTP. Leptomycin B treatment abrogates the acetylated IFI16 translocation (6). The inflammasome complex formation leads into the activation of caspase-1 (7) which in turn cleaves pro-IL-1β and-IL-33 (8), and IL-β is released into the culture supernatant (9). IFI16 acetylation (i) and cytoplasmic redistribution (ii) also activates STING (iii) which phosphorylates IRF-3 (iv) that moves to the nucleus leading into IFN-β gene transcription (v-vi), translation (vii) and IFN-β is secreted into the culture supernatant (viii). Use of p300 inhibitor C-646 and knockdown of p300 does not inhibit the ability of IFI16 to recognize the episomal viral DNA but blocks the acetylation of IFI16 which results in the inhibition of inflammasome formation, IFI16 translocation into the cytoplasm as well as IL-1β and IFN-β production.

Several molecular mechanistic steps of nuclear innate sensing by IFI16 are revealed here (Fig 10). The first step is the recognition of nuclear foreign herpes viral genomes by IFI16 which is independent of acetylation and IFI16 interaction with ASC or STING. This is followed by IFI16’s association with p300 which mediates the acetylation of IFI16. This is a key molecular step common to both of the IFI16 mediated innate responses of inflammasome induction and IFN-β production as IFI16’s acetylation is essential for its interaction with ASC leading into procaspase-1 interaction and activation in the nucleus, interaction with RanGTPase, cytoplasmic translocation and IL-1β induction during KSHV, EBV and HSV-1 infection. Cytoplasmic translocation of acetylated IFI16 is also critical for the activation of STING resulting in the phosphorylation of IRF-3 and IFN-β production (Fig 10).

Crystal structures of overexpressed IFI16 proteins suggest that IFI16 binds to the sugar-phosphate backbone of dsDNA in a non-sequence specific manner with more affinity to superhelix and cruciform DNA [23, 24]. Herpesviral genomes enter the nucleus as a linear, naked dsDNA with nicks and breaks and undergo rapid circularization and chromatinization [25]. Our studies demonstrating that IFI16 recognized the KSHV genome soon after its entry into the nucleus coupled with the fact that this occurs in the absence of acetylation suggests that IFI16 has evolved for rapid recognition of incoming foreign DNA (Fig 10).

Studies with overexpressed proteins suggest that DNA sensing induces filamentous clusters of IFI16 due to homotypic PYD-PYD interactions and cooperative DNA binding that might amplify signals, stabilize IFI16-dsDNA complexes and could act as danger signal [26]. Our studies show such DNA recognition by IFI16 initiates its acetylation process which is essential for the innate immune functions of both inflammasome and interferon responses executed in the cytoplasm and nucleus. Colocalization of reduced levels of acetylated IFI16 with viral genomes (Fig 9) compared to the non-acetylated IFI16 levels suggest that acetylation probably changes the affinity and structure of IFI16 resulting in a dynamic post-genome recognition event of IFI16’s disassociation from the DNA to facilitate its interaction with other proteins and transport into the cytoplasm in a continuous fashion with genomes always occupied with another IFI16 molecule as shown in the KSHV and EBV latently infected cells. This scenario is also supported by observations such as the acetylation of histone prompts its structural extension and charge neutralization resulting in the weakening of DNA-histone interaction [27], and acetylation of KSHV LANA-1 resulting in its dissociation from KSHV genome [28].

Our studies show that acetylation is also critical for IFI16’s transport and interaction with STING, and subsequent IFN-β production in both HSV-1 and KSHV infected cells. Together with our earlier studies demonstrating that ASC is not required for IFN-β production [21, 22] and the absence of IFII6-ASC-procaspase- inflammasome formation and the translocation of acetylated IFI16 in ASC knockdown cells shown here suggested that acetylated IFI16, either alone or in combination with other yet to be identified protein(s), is also relocalized during herpesviral infection resulting in the interaction with STING. Further studies determining whether IFI16 interacts with STING alone or in association with another protein(s) are in progress.

KSHV infection of a PMA stimulated human monocytic THP-1 cell line has been shown to result in IL-1β and IFN-β production by a pathway that is independent of IFI16 [29]. This discrepancy may be due to the fact that KSHV may be undergoing abortive infection in the PMA stimulated cells as has been shown for HSV-1 in these cells [30] and DNA released from the lysosomes is probably recognized by AIM2, c-GAS, and others to stimulate the IL-1β and interferon responses. In contrast, during *in vitro* infection of permissive cells, viral DNA from the capsid enters the IFI16 rich nucleus resulting in the consequences presented by our studies.

Besides its role in inflammasome and interferon induction, IFI16 is also shown to be a transcriptional modulator of normal cells and the mechanisms are poorly defined [19]. Detection of a basal level of IFI16-p300 interaction and acetylated IFI16 in uninfected cells suggest that they may have roles in other cellular functions such as cell cycle regulation and transcription modulation. Increased IFI16-p300 interaction in infected cells suggests that a dynamic process is initiated; however, why the IFI16-p300 interaction increases in the presence of herpesviral DNA and whether IFI16 recruits p300 directly or via its interaction with other proteins needs to be evaluated further. The p300 HAT assay and HDAC assay performed with nuclear and cytoplasmic fractions of KSHV infected HMVEC-d cells and treatment with their respective specific inhibitors revealed the increased p300 activity in the nucleus and not in the cytoplasm, and thus supporting our conclusion that p300 acetylates the IFI16 in the nucleus after viral genome entry into the nucleus (Fig 3B). Simultaneously, the increased activity of HDAC, further demonstrates that the increase in acetylation was not due to decreased activity of HDACs but due to p300 (Fig 3C).

Our recent studies and others suggested that IFI16 promoted the addition of repressive heterochromatin markers and reduced the active euchromatin markers on HSV-1 gene promoters resulting in the reduced binding of transcription factors and RNA pol II [22]. Whether the regulatory functions of these genes are independent or dependent on the acetylation of IFI16 needs to be determined which is beyond the scope of the present studies.

Increased IFI16 oligomerization in KSHV infected cells due to acetylation suggests that acetylation mediated structural changes in IFI16 probably favors its increased binding with multiple ASC molecules leading into inflammasome assembly. Similarly, ligand mediated NLRC4 phosphorylation has been shown to be crucial for inflammasome activation [31]. In addition, ASC phosphorylation at the CARD domain has been shown to be critical for speck like aggregation and for NLRP3 and AIM2 mediated inflammasomes activation [32]. Though IFI16 has also been shown to be phosphorylated by pUL97 of HCMV that relocalizes IFI16 to the cytoplasm [33], its role in the context of innate immunity has not been evaluated. The Li et al., [15] studies with total cell extracts from human CEM-T lymphoblast-like cells identified six phosphorylation and nine acetylation sites on endogenous IFI16. Which of these sites undergo modifications during the recognition of nuclear viral genomes needs to be examined further and is beyond the scope of the present study. In addition, using uninfected U2OS transfected with DNA, Li et al., [15] demonstrated that acetylation at the NLS motifs of IFI16 results in the cytoplasmic retention of newly synthesized IFI16 by inhibiting nuclear import, and p300 regulated the cytoplasmic IFI16 acetylation during transfection of DNA. As the NLS motif is essential for IFI16 to enter the nucleus, studies with NLS mutants were not possible in our experimental approaches since these mutants IFI16 will stay in the cytoplasm and will not detect the herpes virus genome. Further understanding of IFI16’s modifications will shed additional light on its role in host innate responses as well as its cell cycle and transcription regulatory functions.

In summary, our studies demonstrate that the post-genome recognition event of IFI16’s acetylation by histone acetyltransferase p300 is required for the IFI16-mediated innate immune responses of inflammasome induction, IL-1β and interferon-β production during herpesvirus infections.

## Materials and Methods

### Cells

HMVEC-d and HFF cells (Clonetics, Walkersville, MD), TIVE, TIVE-LTC, BCBL-1, BJAB-KSHV, Raji, LCL, BJAB, and Ramos cells were grown as described before (11, 12,13, 14). Cells were routinely tested for mycoplasma and only mycoplasma free cells were used for experiments.

### Reagents

Protein A-Sepharose and Protein G-Sepharose CL-4B Fast Flow beads were from GE Healthcare Bio-Sciences Corp., Piscataway, NJ. Cyto Nuclear extract kit was from Active Motif, Carlsbad, CA. Trichostatin A (TSA), Leptomycin B (LPT), and nicotinamide were from Sigma-Aldrich. The CytoTox 96 non-radioactive cytotoxicity kit was from Promega, Madison, WI. SlowFade Gold Antifade reagent with DAPI was from Life Technologies. Verikine human IFN-β ELISA kit was from PBL Assay Science, Piscataway Township, NJ. IL-1β ELISA kit was from RayBiotech, Inc. Norcross, GA. The FLICA 660 Caspase-1 Assay kit was from Immunochemistry Technologies, Bloomington, MN. P300 and HDAC activity assay kits were from BioVision Inc., Milpitas, CA.

### Antibodies (Table 1)

Mouse monoclonal anti-IFI16, rabbit polyclonal anti-p300, mouse monoclonal anti-IL-33 and rat polyclonal anti-BrdU antibodies were from Santa Cruz Biotechnology Inc., Santa Cruz, CA. Rabbit anti-BrdU antibody was from Rockland Inc., Gilbertsville, PA. Mouse monoclonal anti-β-actin and tubulin antibodies plus rabbit anti-human IFI16 antibodies were from Sigma-Aldrich. Mouse monoclonal anti-human IL-1β and caspase-1 antibodies were from R&D Systems, Minneapolis, MN, and Invitrogen, Carlsbad, CA, respectively. Goat polyclonal antibody against human ASC was from RayBiotech. Mouse monoclonal antibody against ASC was from MBL International, Woburn, MA. Mouse anti-human TATA binding protein (TBP), rabbit anti-human Ran and mouse anti-IRF-3 antibodies were from Abcam Inc., Cambridge, MA. Rabbit anti-cyclin B1, rabbit monoclonal anti-STING,-p-IRF-3,-histone H2B and H3 antibodies were from CST, Danvers, MA. Anti-rabbit, goat and mouse antibodies linked to horseradish peroxidase, Alexa Fluor-488, -594 and -647 were from KPL Inc., Gaithersburg, MD, or Molecular Probes, Eugene, OR. Anti-Mouse IgG (heavy-chain spcificity)-HRP conjugate goat antibody was from Alpha Diagnostics Intl. Inc. San Antonio, TX. Anti-mouse tagged with IR Dye 680RD secondary antibodies were from LI COR Biotechnology, Lincoln, NE.

### KSHV and BrdU-KSHV

Induction of the lytic cycle in BCBL-1 cells by phorbol ester, supernatant collection, and virus purification were described previously [11, 18]. For generating 5-bromo-2-deoxyuridine (BrdU) incorporated KSHV genome, BrdU labeling reagent (Life Technologies) was added to the culture medium in a 1:100 (v:v) ratio (from the supplied stock) [34]. KSHV DNA was extracted, copy numbers quantitated by real-time DNA-PCR, and infection was done with 30 genome copies/cell [11].

### KSHV infection in the presence of inhibitors

HMVEC-d or HFF cells pre-starved for 2 h in the presence or absence of inhibitors such as C-646, leptomycin or cycloheximide, washed, left uninfected or infected with 30 DNA copies/cell in serum free medium for different time points, washed and incubated in complete medium in the presence or absence of inhibitors for different time periods. KSHV positive BCBL-1, BJAB-KSHV and TIVE-LTC cells and uninfected BJAB and TIVE cells were incubated with inhibitors for different time points.

### HSV-1 infection

The KOS strain of HSV-1 was produced and titer determined by plaque assay on Vero cells as described [14]. To generate BrdU labelled genome HSV-1, we added BrdU labeling reagent (Life Technologies) to the culture medium at 8 h, 24 h and 48 h post infection in a 1:100 (v:v) ratio (from the supplied stock). HFF cells were starved for 2 h in the presence or absence of C-646, washed and infected with HSV-1 at a multiplicity of infection (MOI) of 1 PFU/cell (~25 genome copies/cell) in serum-free DMEM with or without C-646 for different times, washed with PBS, and incubated in DMEM supplemented with 2% FBS for different time points.

### Cytotoxicity assay

A non-radioactive cytotoxicity assay was performed according to the manufacturer’s protocol (Promega) to evaluate the various inhibitors used in this study. Briefly, HMVEC-d, BCBL-1, TIVE, TIVE-LTC and HFF cells cultured in 12 well plates were incubated with DMSO or varying concentrations of C646 in DMSO for 4 and 24 h in their respective complete media. 100 μl of culture medium of each group was taken carefully and treated with 10 μl of lysis solution (Promega) and incubated for 45–60 min in a 37°C incubator with 5% CO2. Plates were then centrifuged at 1,000 RPM for 3 min and 50 ul samples were transferred carefully into separate 96 well plates with 3 positive control wells of LDH (supplied in the kit). 50 μl of substrate was added to each well of the plate, incubated at RT for 15–30 min, and read at 490 nm using an ELISA reader. The positive control was considered as 100% cytotoxic.

### Measurement of KSHV nuclear entry by real-time DNA polymerase chain reaction (PCR)

HMVEC-d were starved for 2 h with or without C-646 and either left uninfected or infected with KSHV (30 DNA copies/cell) at 37°C for 2 h. These cells were washed, treated with trypsin-EDTA to remove non-internalized virus, and incubated for varying times of infection [18]. Nuclei were isolated using a Nuclei EZ Prep isolation kit (Sigma) according to the manufacturer’s instructions. Briefly, cells were lysed on ice for 5 min with a mild lysis buffer (Sigma), and nuclei were concentrated by centrifugation at 500x*g* for 5 min. Cytoskeletal components loosely bound to the nuclei were removed from the nuclear pellet by a repeat of the lysis and centrifugation procedures as described previously [11]. DNA was extracted from isolated nuclei using a DNeasy kit (Qiagen, Germantown, MD). Internalized nuclear KSHV DNA was quantitated by amplification of the ORF73 gene by real-time DNA PCR [2]. The KSHV ORF73 gene cloned in the pGEM-T vector (Promega) was used for the external standard. The *CT* values were used to generate the standard curve and to calculate the relative copy numbers of viral DNA in the samples.

### Measurement of KSHV gene expression by real-time reverse transcription (RT) PCR

Total RNAs from KSHV infected or uninfected HMVEC-d cells in the presence or absence of inhibitors were prepared using an RNeasy kit (Qiagen). To quantitate viral gene expression, total RNA was subjected to real-time RT-PCR using ORF73 gene-specific primers and TaqMan probes. A standard curve using the *CT* values of different dilutions of *in vitro*-transcribed transcripts was used to calculate relative copy numbers of the transcripts. These values were normalized to those for GAPDH (glyceraldehyde- 3-phosphate dehydrogenase) control reactions. To obtain p values between DMSO, C-646, Leptomycin-B and cycloheximide treated cells, an unpaired Student’s *t* test was used.

### KSHV and EBV infection in PBMC

For *de novo* infection, peripheral blood mononuclear cells (PBMCs) were obtained from the University of Pennsylvania CFAR Immunology Core, and 1X10^7^ PBMCs were either left uninfected or infected by KSHV or EBV as previously described [13]. Briefly, PBMCs were infected with KSHV or EBV in 1 ml of RPMI 1640 medium supplemented with 10% FBS and 5 ng/ml of polybrene (Sigma-Aldrich), incubated for 4 h at 37°C (time point 0), and infected and uninfected cells were centrifuged at 1,200*g* for 5 min. Cells were washed twice with RPMI medium, resuspended and cultured in six-well plates at 37°C in fresh RPMI medium with 10% FBS. At 24 h p.i., the cells were washed twice with 1X PBS and spotted on slides.

### Protein cross-linking

HMVEC-d cells pre-starved for 2 h in the presence or absence of C-646 were washed, uninfected or KSHV infected for 2 h, washed, and incubated with complete medium with or without C-646 for 24 h. BJAB and BCBL-1 cells were treated with C-646 for 24 h. These cells were lysed in HEPES-lysis buffer (100 mM NaCl, 40 mM HEPES [pH 7.5], 05% (v/v) glycerol, 0.1% (v/v) Nonidet P-40 [NP-40] supplemented with PIC). The lysates were cross-linked with 5 mM glutaraldehyde for 10 min, reaction terminated with the addition of 10 μl of 1M Tris-HCL (pH 8.0), samples boiled in SDS buffer, and analyzed by western blot to detect IFI16 oligomerization.

### Si-RNA transfection

All Si-RNA oligonucleotides for ASC and p300 were from Santa Cruz Biotechnology, Inc. STING Si-RNA (smart pool: Si genome TMEM173, cat. No. M-024333-00-0010) were from GE-Dharmacon (Fisher-Scientific, Pittsburgh, PA) Primary HMVEC-d cells were transfected with Si-RNA using a Neon transfection system (Invitrogen) according to the manufacturer’s instructions. Briefly, subconfluent cells were detached from the culture flasks, washed once with PBS and resuspended in buffer R (Invitrogen) at a density of 1X10^7^ cells/ml. 10 μl of the cell suspension was gently mixed with control Si-RNA or 100 pmol of target specific Si-RNA and then microporated at room temperature using a single pulse of 1,350 V for 30 ms. After microporation, cells were distributed into pre-warmed complete medium and placed at 37°C in a humidified 5% CO2 atmosphere. At 48 h post-transfection, cells were infected with KSHV as described earlier and incubated for 24 h, whole cell lysates using NETN or cytoplasmic/nuclear extract buffers were isolated, knockdown efficiency evaluated by western blotting, and then subjected to co-IP and western blotting.

### Co-immunoprecipitation (co-IP)

KSHV infection-induced protein acetylation and other protein-protein interactions were evaluated by co-IP experiments using equal amounts of WCL as well as cytoplasmic and nuclear lysates. The lysates were first incubated for 2 h with 15 μl of Protein A/G sepharose beads and then the pre-cleared lysates incubated for 2 h with immunoprecipitating antibody (anti-acetylated lysine,-IFI16,-ASC,-caspase-1,-p300 antibodies) at 4°C. The immune complexes were captured using 15 μl of Protein A/G-Sepharose beads, washed 4 times with lysis buffer, 3 times in PBS, boiled with SDS-PAGE sample buffer, resolved by 10% SDS-PAGE, and subjected to western blotting.

### Immunofluorescence microscopy assay (IFA)

HMVEC-d cells grown on fibronectin-coated 8 well chamber glass slides for 48 h were serum-starved in the presence or absence of inhibitors for 2 h, washed and then either left uninfected or infected with KSHV (30 DNA copies/cell) for 2 h. Cells were washed with PBS, incubated in complete medium for various time points, washed, fixed in 4% paraformaldehyde for 10 min and permeabilized with 0.2% Triton X-100 for 5 min. BJAB, BCBL-1 and BJAB-KSHV suspension cells treated or untreated with C-646 for 24 h were fixed and permeabilized with pre-chilled acetone. Cells were washed and blocked with Image-iT FX signal enhancer (Invitrogen) for 20 min at RT, and incubated with specific antibodies diluted in 2% BSA for 2 h at 37°C. After washing, cells were incubated with Alexa-Fluor conjugated appropriate secondary antibodies for 1 h at 37°C, washed, mounted in DAPI, imaged with Nikon Eclipse 80i fluorescence microscope and analyzed by Nikon Elements software.

### *In situ* proximity ligation assay (PLA) microscopy

A DuoLink PLA kit from Sigma-Aldrich was used to detect protein–protein interactions as per manufacturer’s protocol. Cells were infected with KSHV (30 DNA copies/cell) or HSV-1 (1 PFU/cell; ~25 genome copies/cell), fixed and permeabilized as described in the IFA section and blocked with DuoLink blocking buffer for 30 min at 37°C. These cells were incubated with target specific primary antibodies diluted in DuoLink dilution buffer. After washing, the cells were incubated for another 1 h at 37°C with species specific PLA probes (PLUS and MINUS) under hybridization conditions and in the presence of 2 additional oligonucleotides to facilitate hybridization of PLA probes if they were in close proximity (<40 nm). A ligation mixture and ligase were then added to join the two hybridized oligonucleotides to form a closed circle. Amplification solution was added to generate a concatemeric product extending from the oligonucleotide arm of the PLA probe. Finally, a detection solution consisting of fluorescently labeled oligonucleotides was added, and the labeled oligonucleotides were hybridized to the concatemeric products. The signal was detected as a distinct fluorescent dot in the Texas red or FITC green channel and analyzed by fluorescence microscopy. Negative controls consisted of samples treated as described but with only secondary antibodies. In some experiments, BrdU staining was performed before PLA to detect viral genome in the infected cells.

### Active caspase-1 assay

BJAB and BCBL-1 cells were subjected to the FLICA 660-YVAD-FMK Caspase-1 assay to detect the active caspase-1 in C-646 treated or untreated cells. The BCBL-1 cells were incubated with C-646 (p300 inhibitor) overnight, washed and FLICA 660-YVAD-FMK caspase-1 detection reagent was applied for 1 h to stain the cells with active caspase-1 in untreated or C-646 treated cells. The cell permeable FLICA 660-YVAD-FMK caspase-1 detection reagent efficiently diffuses into cells and irreversibly binds to activated caspase-1 enzymes. Cells without active caspase-1 have a non-fluorescent status after the wash step. The cells were fixed into the fixation media provided by the manufacturer. The cells were washed to remove the unbound FLICA 660 and subjected to flow cytometry (LSRII, BD Biosciences) at the Flow Cytometry Facility at Rosalind Franklin University of Medicine and Science.

### Western blot analysis

The whole cell protein lysates (WCL) from uninfected and KSHV infected cells were prepared using NETN lysis buffer (100 mM NaCl, 20 mM Tris-HCl [pH 8.0], 0.5 mM EDTA, 0.5% (v/v) Nonidet P-40 [NP-40]) supplemented with 10 μM TSA, 5 mM nicotinamide and protease inhibitor cocktail. Cells were incubated on a rocker at 4°C for 15 min and sonicated at 40 amplitude three times with pulses of 15 seconds on and 10 seconds off. Lysates were clarified by centrifugation for 15 min at 4°C at 15000 x g. The nuclear and cytoplasmic extracts were prepared following the manufacturer’s procedure (Active Motif, Carlsbad, CA). Equal amounts of samples were resolved by 10–20% SDS-PAGE, subjected to western blot, immunoreactive bands developed by enhanced chemiluminescence reaction (NEN Life Sciences Products, Boston, MA), and the bands scanned and quantitated using an AlphaImager (Alpha Innotech Corporation, San Leonardo, CA). The bands were scanned and quantitated using FluorChemFC2 software and an AlphaImager system (Alpha Innotech Corporation, San Leonardo, CA). To detect the tubulin in IP samples, secondary anti-mouse (IRdye 680 labelled) antibody was used and immunoblots were visualized by using the LI-COR Odyssey system

### IFN-β and IL-1β detection assay

The HFF or HMVEC-d cells in 6 well plates were starved and infected with HSV-1 or KSHV, respectively, for 30 min or 2 h with or without C-646 and incubated for 6 h. Culture supernatants were centrifuged and subjected to ELISA for detection of IFN-β. HMVEC-d cells were starved for 2 h and infected with KSHV-1 with or without C-646 for 2 h, washed and incubated for 24 h and culture supernatant was used to detect IL-1β by ELISA performed as per manufacturer’s instructions. Briefly, the culture supernatants and standards were incubated in the pre-coated wells for 1 h, washed with the washing buffer provided in the kit and probed with IFN-β antibody for 1 h. These wells were washed, incubated with HRP tagged antibodies for 1 h, washed, incubated with substrate (TMB) for 15 min and the reaction was terminated with a stop solution. After 5 min, readings were taken at 450nm and calculations done using a standard curve.

### p300 histone acetyltransferase activity assay

The p300 histone acetyltransferase activity was measured using the p300 HAT fluorometric assay kit from Biovision (Mountain View, CA) as per the manufacturer’s instructions with slight modifications. Briefly, HMVEC-d cells were either left uninfected or infected with KSHV for 24 h in the presence or absence of C-646, and cytoplasmic and nuclear fractions were isolated. From each group 5 μg protein was incubated with the p300 substrate (H3 peptide and acetyl CoA) at 30°C for 30 min in a 96 well plate. The reaction was stopped by adding pre-chilled isopropyl alcohol followed by addition of thiol detecting probe and incubated at room temperature for 15 min. The plate was read for fluorescence at Ex/Em = 392/482 nm in a plate reader (BioTek, Winooski, VT).

### HDAC activity assay

The HDAC activity was measured using a fluorometric assay kit from BioVision (Mountain View, CA) as per the manufacturer’s instructions. Protein samples were prepared as in the p300 activity experiment of HMVEC-d cells infected with KSHV for 24 h in the presence or absence of Tricostatin-A (TSA). From each group 10 μg of nuclear and cytoplasmic proteins were mixed in HDAC assay buffer, the fluorometric substrate was added to each well of the 96 well plate and incubated at 37°C for 30 min. The reaction was stopped by adding Lysine developer and mixed well followed by incubation at 37°C for 30 minutes. The samples were analyzed in a fluorescence plate reader (Ex/Em = 350–380/440–460 nm).

### Chromatin Immunoprecipitation assay (ChIP)

To detect the direct association of IFI16 with viral DNA, Chromatin Immunoprecipitation (ChIP) assay was performed as per manufacturer’s instructions. Briefly, untreated BCBL-1 cells (3 x 10^7^) or cells treated with 1μM C-646 for 24 h were fixed with 1% (v/v) methanol-free formaldehyde in fixing buffer for 5 min, crosslinking was quenched using quenching buffer for 5 min at RT, cells were washed twice with cold PBS then incubated in lysis buffer to break the cell membrane. Intact nuclei were collected by centrifugation at 1,700 x g for 5 min at 4°C, resuspended in shearing buffer containing protease inhibitors and chromatin shearing performed on an AFA (Adaptive Focused Acoustics) ultrasonicator (Covaris M220).

Following chromatin shearing, ChIP was performed as described previously (22). Briefly, cellular debris was cleared from the sheared chromatin by centrifugation and the supernatant was incubated overnight at 4°C with 1.5 μg of IFI16 antibody. Samples were incubated in ChIP grade Protein G Magnetic Beads for 2 h at 4°C to collect immune complexes and then washed successively with low salt wash buffer (0.1% SDS; 1% Triton X-100; 2 mM EDTA; 20 mM Tris, [pH 8.1]; 150 mM NaCl), then high salt wash buffer (0.1% SDS; 1% Triton X-100; 2 mM EDTA; 20 mM Tris, [pH 8.1]; 500 mM NaCl), and then LiCl wash buffer (0.25 M LiCl; 1% NP-40; 1% deoxycholate; 1 mM EDTA; 10 mM Tris, [pH 8.1]). DNA-protein complexes were eluted in 1% SDS prepared in 0.1 M NaHCO_3_. Crosslinking was reversed by adding 1 μL RNase A and NaCl (0.3 M) and incubating at 65°C for 5 h. Protein was removed by incubating lysate with proteinase K at 55°C for 1 h. Subsequently, DNA was purified using the Wizard SV Genomic DNA Purification System (Promega) and resuspended in nuclease-free water. Real-time PCR was performed with the following KSHV genome (NCBI Reference NC_009333.1) specific primers, Primer Set 1: CAAGGTTAAAGTGGGTTTGCTG, GGTTATTGGCCGTTTCTGTTTC and Primer Set 2: GCGTAATTACTTCCGAGACTGA, TTAACTCCACTTTGCA CCAAAC. As a cellular control, human positive control primer set GAPDH-2 from Active Motif was used. ChIP results are represented as fold enrichment over IgG control.

### Statistical analysis

Results are expressed as means ± SD of at least three independent experiments (n≥3). The p value was calculated using a Student’s T test. In all tests, p*<0*.*05* was considered statistically significant.

## Supporting Information

S1 Fig Immunofluorescence analysis of augmentation of IFI16’s acetylation in the nucleus and its redistribution to the cytoplasm during *de novo* infection of HMVEC-d cells. **(A)** IFI16 redistribution kinetics during *de novo* infection. Serum starved HMVEC-d cells (2 h) were uninfected (UI) or infected with KSHV (30 DNA copies/cell) for 30 min or 2 h, the 2 h cells washed and incubated for various time points. Cells were fixed, permeabilized and blocked with Image-iT signal enhancer, reacted with anti-IFI16 antibodies, washed and probed with Alexa Fluor-488 secondary antibodies. Nuclei were stained with DAPI. The boxed areas are enlarged and red arrows indicate the cytoplasmic IFI16. **(B)** Acetylated IFI16 redistribution kinetics during *de novo* infection. Uninfected and KSHV infected HMVEC-d cells as described above were processed for IFA, reacted with anti-IFI16 and anti-acetylated lysine antibodies, washed and reacted with Alexa Fluor-488 and Alexa Fluor-594 conjugated secondary antibodies. Nuclei were stained with DAPI and boxed areas are enlarged. The yellow arrows indicate the cytoplasmic IFI16. The red arrows indicate the acetylated IFI16 in the nucleus and white arrows indicate the acetylated IFI16 in the cytoplasm.
(TIF)

S2 Fig Cytotoxicity screening of C-646 (p300 inhibitor) treatment and its effect on KSHV infectivity and target the acetylation of proteins in the infected cells. The cytotoxicity of various concentrations of C-646 was determined using a Promega cytotoxicity kit, by measuring the released LDH in culture supernatants of **(A)** BCBL-1 and **(B)** HMVEC-d cells. **(C)** HMVEC-d cells serum-starved in the presence or absence of 1 μM C-646 for 2 h were washed and infected with KSHV for 2 h. DNA isolated from the nucleus of infected cells was evaluated for nuclear delivery of KSHV genome using real-time-DNA PCR. The nuclear viral DNA copy number was calculated using a standard curve generated from known concentrations of the ORF73 gene. **(D, E and F)** HMVEC-d cells serum-starved with or without 1 μM C-646 for 2 h were washed, infected with KSHV for 2 h, washed, and incubated with complete medium in the presence or absence of 1 μM C-646 for 24 h. **(D)** Cells were fixed, permeabilized, blocked with Image-iT FX signal enhancer, incubated with mouse anti-KSHV LANA-1 antibody and then probed with Alexa Fluor-488 conjugated secondary antibodies. White arrows indicate the LANA-1 dots in the nucleus of the infected cells and red arrows indicate uninfected cells. **(E)** The LANA-1 dots per infected cell were enumerated from at least 5 different fields with a minimum 10 cells and results plotted as a bar graph. **(F and G)** HMVEC-d cells serum-starved in the presence or absence of 1 μM C-646 for 2 h were either left uninfected or infected with KSHV (30 DNA copies/cell) for 2 h and incubated for 24 h in complete medium with or without 1 μM C-646. **(F)** Equal quantities of total cell lysate proteins in NETN buffer were western blotted with anti-acetylated antibody. **(G)** Equal quantities of whole cell lysates from the 24 h time point described above were IP-ed with anti-acetylated lysine antibody and western blotted for H2B. Total H2B and tubulin were used as input and loading controls, respectively.
(TIF)

S3 Fig Induction of acetylation in HFF cells during *de novo* KSHV infection. **(A)** HFF cells serum-starved in the presence or absence of 1 μM C-646 for 2 h were infected with KSHV (30 DNA copies/cell) for 2 h, washed, and incubated with complete medium for 24 h with or without 1 μM C-646. Equal amounts of total protein lysates in NETN-lysis buffer were IP-ed with anti-acetylated lysine antibodies and immunoblotted for IFI16. Total IFI16 and tubulin were used as loading controls. **(B and C)** HFF cells serum-starved in the absence or presence of 1 μM C-646 for 2 h were either left uninfected or infected with KSHV for 2 h, washed, cultured in complete medium for 24 h with or without 1 μM C-646 and subjected to PLA with anti-acetylated lysine and anti-IFI16 antibodies (B) or with anti-IFI16 mouse and rabbit antibodies (C). DAPI was used to stain the nucleus. Cytoplasmic and nuclear acetylated IFI16 in panel (B) denoted by white and yellow arrows, respectively. White and yellow arrows in panel (C) depict cytoplasmic and nuclear IFI16, respectively.
(TIF)

S4 Fig IFI16 acetylation and its cytoplasmic redistribution in KSHV latently infected B and endothelial cells. **(A)** BJAB (KSHV-) and BCBL-1 (KSHV+) cells were untreated or treated with 1 μM C-646 for 24 h, and WCL proteins in NETN buffer were IP-ed with anti-acetylated lysine antibodies and western blotted for IFI16. **(B)** The nuclear and cytoplasmic extracts from untreated BCBL-1 cells or cells treated with 1 μM C-646 for 4 and 24 h were western blotted for IFI16, TBP and tubulin. **(C)** BJAB and BCBL-1 cells in the presence or absence of 1 μM C-646 (24 h) were tested by PLA with anti-IFI16 and anti-acetylated lysine antibodies. White arrows and yellow arrows indicate cytoplasmic and nuclear acetylated IFI16, respectively. **(D)** BJAB and BCBL-1 cells left untreated or treated with 1 μM C-646 (24 h) were tested by PLA with anti-IFI16 mouse and rabbit antibodies. White and yellow arrows indicate cytoplasmic and nuclear IFI16, respectively. **(E)** WCL proteins in NETN buffer were IP-ed with anti-acetylated lysine antibodies and western blotted for IFI16. **(F and G)** TIVE and TIVE-LTC cells untreated or treated with 1 μM C-646 for 24 h were analyzed by PLA. (F) PLA using anti-IFI16 and anti-acetylated lysine antibodies. White arrows and yellow arrows indicate cytoplasmic and nuclear acetylated IFI16, respectively; (G) Anti-IFI16 mouse and rabbit antibodies. White arrows and yellow arrows indicate cytoplasmic and nuclear IFI16, respectively.
(TIF)

S5 Fig PLA analysis of IFI16 acetylation in KSHV and EBV-infected primary B cells and EBV latently infected cells. Human B cells (PBMCs) infected with KSHV or EBV for 24 h **(A)** and EBV negative Ramos and EBV positive RAJI and LCL cells **(B)** were fixed in acetone, blocked in Duolink blocking buffer, reacted with anti-IFI16 and anti-acetylated lysine antibodies and subjected to PLA. The acetylated IFI16 is represented as red dots. Yellow and white arrows depict nuclear and cytoplasmic acetylated IFI16, respectively. The right most panels show their corresponding bright field images. The images shown here are representative of three independent experiments. Magnification: 60X.
(TIF)

S6 Fig PLA analysis of IFI16 acetylation in HMVEC-d cells infected with Vaccinia virus. HMVEC-d cells were serum-starved in the absence or presence of 1 μM C-646 for 2 h, then either left uninfected or infected with Vaccinia virus (5 PFU/cell) for 2 h, washed, cultured in complete medium for 8 h with or without 1 μM C-646 and subjected to PLA with anti-acetylated lysine and anti-IFI16 antibodies **(A)** or with anti-IFI16 mouse and rabbit antibodies **(B).** DAPI was used to stain the nucleus. The red inset is enlarged in the right most panels. The images shown here are representatives of three independent experiments. Magnification: 60X.
(TIF)

S7 Fig Cytotoxicity screening of Leptomycin B and Cycloheximide on and their effect on KSHV infectivity in HMVEC-d cells. **(A and B)** The toxicity of various concentrations of Leptomycin B (LPT) and Cycloheximide (CHX) were evaluated in HMVEC-d cells. **(C and D)** HMVEC-d cells were serum- starved for 2 h in the presence or absence of 50 nM Leptomycin B or 200 μg/ml Cycloheximide, washed, left uninfected or infected with KSHV (30 DNA copies/cell) for 2 h and nuclei associated viral DNA copy numbers were estimated by real-time DNA PCR for the ORF73 gene. Results shown represent mean ± SD for three experiments. **(E and F)** HMVEC-d cells treated or untreated with 50 nM LPT or 200 μg/ml Cycloheximide during 2 h starvation were washed, infected with KSHV for 2 h, washed and incubated for 24 h in complete medium with or without inhibitors. RNA was subjected to qRT-PCR for ORF73 transcripts. Results shown represent mean ± SD for three experiments.
(TIF)

S8 Fig IFI16-ASC interaction and inflammasome activation in *de novo* KSHV infected HMVEC-d cells and latently infected BCBL-1 cells in the presence of C-646. **(A)** HMVEC-d cells serum-starved with or without 1 μM C-646 were infected with KSHV for 2 h, washed, incubated in complete medium for 24 h in the presence or absence of 1 μM C-646 and IFA for IFI16 and ASC performed. DAPI was used to stain the nucleus. Insets in the merged panels are enlarged in the right most panels. The white and red arrows indicate the IFI16-ASC colocalization spots in the cytoplasm and nucleus, respectively. Magnification: 60X. **(B)** BCBL-1 cells untreated or treated with 1 μM C-646 were reacted with mouse anti-IFI16 and goat anti-ASC antibodies and then probed with Alexa Fluor-488 and Alexa Fluor-594 conjugated secondary antibodies, respectively. Nucleus was stained with DAPI. The white arrows depict the cytoplasmic IFI16-ASC colocalization. Images shown here are representative of three independent experiments. Magnification: 60X. **(C)** Percent live cells in BJAB and BCBL-1 cells in the absence or presence of 1 μM C-646 for 24 h were determined prior to FACS using the Trypan blue exclusion method. **(D)** The BJAB and BCBL-1 cells with active caspase-1 were enumerated using a FLICA 660-YVAD-FMK staining kit. Cells were washed in PBS, incubated with FLICA 660-YVAD-FMK dye, unbound dye removed by washing with PBS and analyzed by FACS. **(E)** BCBL-1 cells untreated or treated with 1 μM C-646 were examined for active caspase-1 using the above described kit and flow cytometry analysis was performed. **(F)** Percent active caspase-1 in total cell population of BJAB and BCBL-1 cells treated or untreated with 1 μM C-646 was enumerated using the data from FACS analysis and presented in the form of a bar graph.
(TIF)

S9 Fig Effect of acetylation inhibitor C-646 on IFI16 mediated HSV-1 genome recognition. The cytotoxicity of various concentrations of C-646 was determined using a Promega cytotoxicity kit, by measuring the released LDH in culture supernatant of **(A)** HFF cells. **(B)** Vero cells serum-starved in the presence or absence of 1 μM C-646 for 2 h were washed and infected with HSV-1 (1 PFU/cell MOI) for 2 h then plaque assay was performed to assay the effect of C-646 on HSV-1 infectivity and production. **(C)** HFF cells pre-incubated with or without 1 μM C-646 for 2 h were washed, infected with BrdU genome labeled HSV-1 (1PFU/cell) for 2 h, washed and incubated with or without 1 μM C-646 for 4 h. Cells were subjected to PLA with anti-IFI16 and anti-BrdU antibodies. Boxed areas are enlarged. PLA spots indicated by the representative white arrows indicate the association between BrdU labeled HSV-1 genome and IFI16. The images shown here are representative of three independent experiments. Magnification: 60X. **(D)** PLA spots (C) from 10 fields with at least 3–4 cells/field were quantitated and presented here as a bar graph.
(TIF)
