# Supplementary figures and images for: Herpesvirus Genome Recognition Induced Acetylation of Nuclear IFI16 Is Essential for Its Cytoplasmic Translocation, Inflammasome and IFN-β Responses
Source: PLoS Pathog. 2015 Jul 2;11(7):e1005019. doi: 10.1371/journal.ppat.1005019 (PMC4489722; doi:10.1371/journal.ppat.1005019)

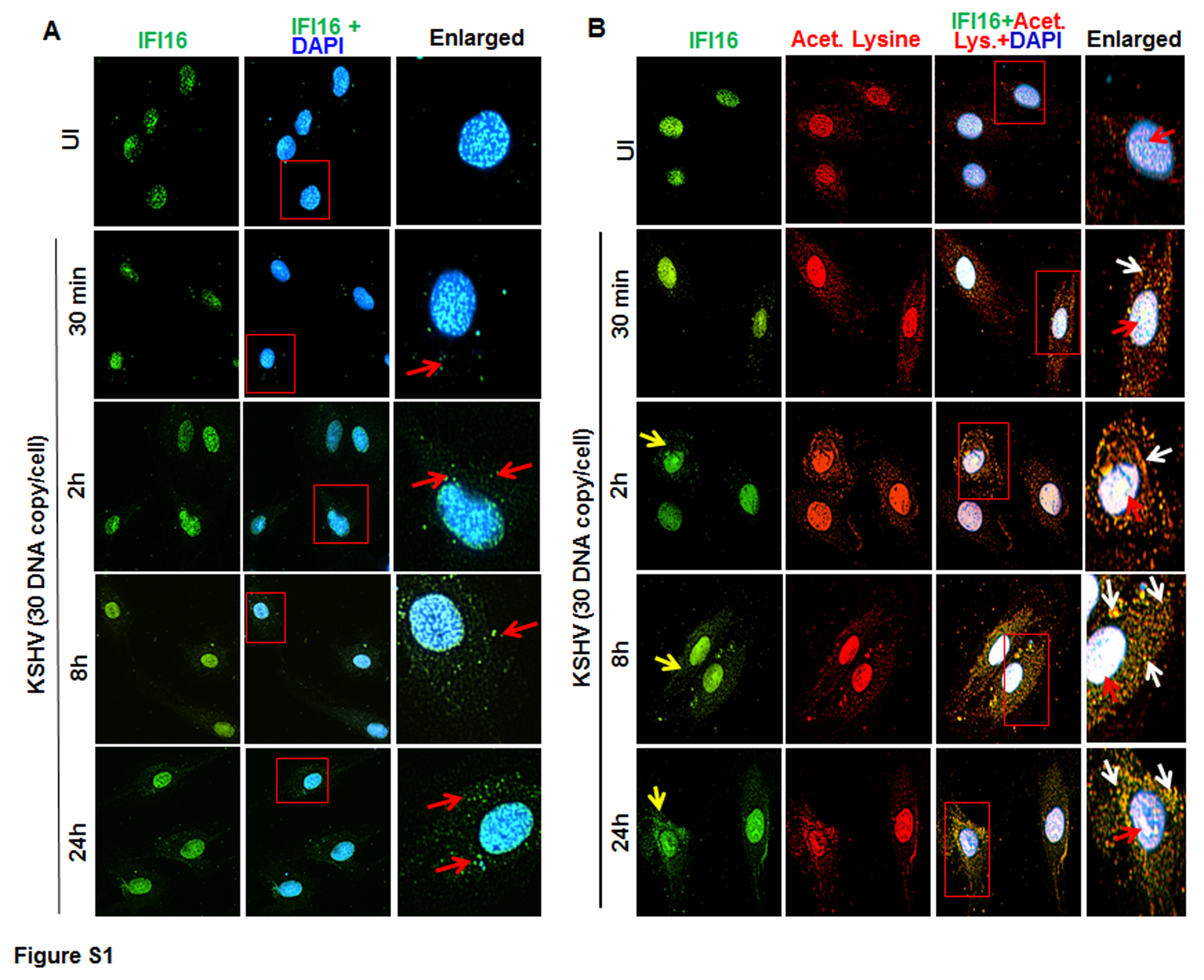

Supplement: S1 Fig — (A) IFI16 redistribution kinetics during de novo infection. Serum starved HMVEC-d cells (2 h) were uninfected (UI) or infected with KSHV (30 DNA copies/cell) for 30 min or 2 h, the 2 h cells washed and incubated for various time points. Cells were fixed, permeabilized and blocked with Image-iT signal enhancer, reacted with anti-IFI16 antibodies, washed and probed with Alexa Fluor-488 secondary antibodies. Nuclei were stained with DAPI. The boxed areas are enlarged and red arrows indicate the cytoplasmic IFI16. (B) Acetylated IFI16 redistribution kinetics during de novo infection. Uninfected and KSHV infected HMVEC-d cells as described above were processed for IFA, reacted with anti-IFI16 and anti-acetylated lysine antibodies, washed and reacted with Alexa Fluor-488 and Alexa Fluor-594 conjugated secondary antibodies. Nuclei were stained with DAPI and boxed areas are enlarged. The yellow arrows indicate the cytoplasmic IFI16. The red arrows indicate the acetylated IFI16 in the nucleus and white arrows indicate the acetylated IFI16 in the cytoplasm. (TIF) [file ppat.1005019.s001.tif]

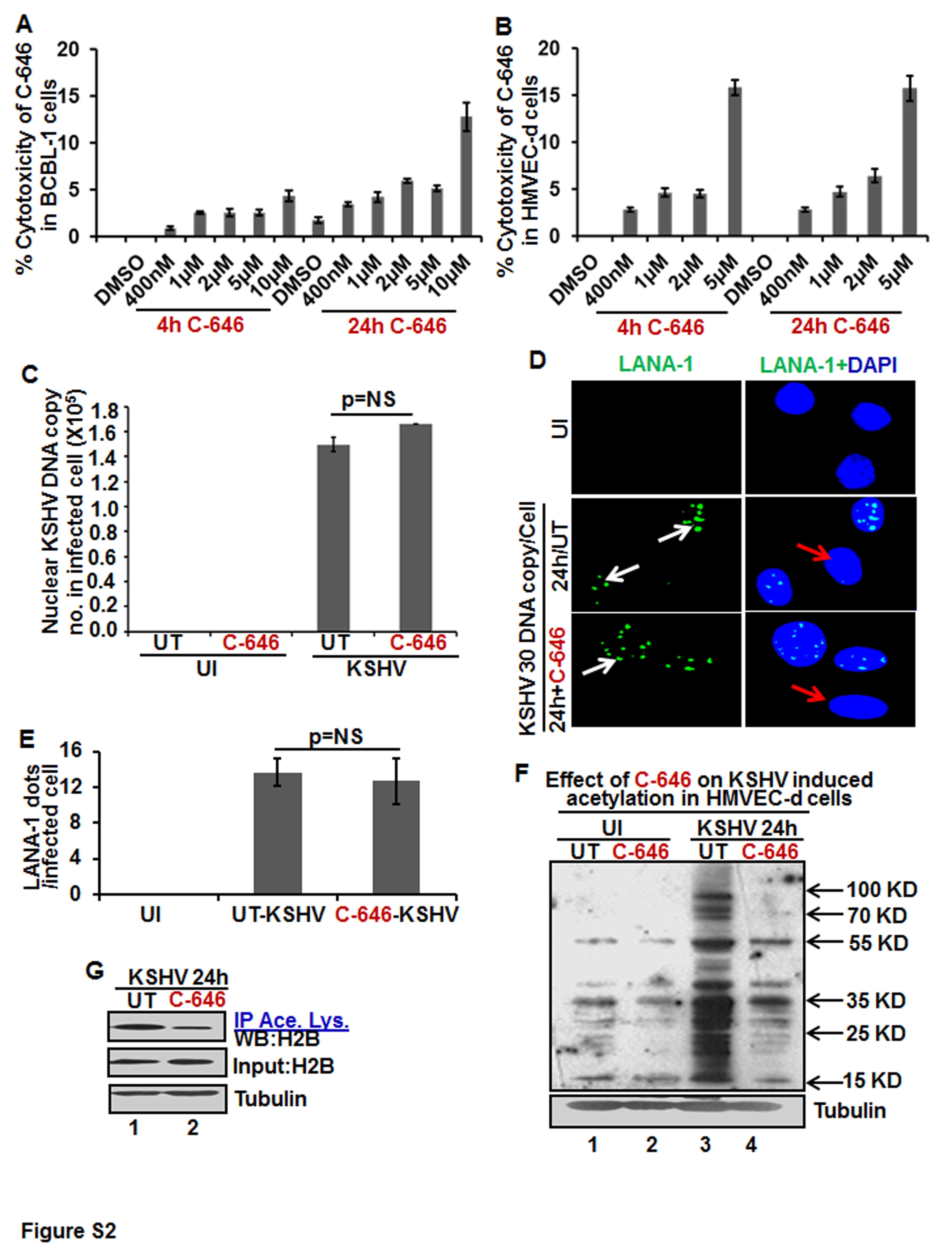

Supplement: S2 Fig — The cytotoxicity of various concentrations of C-646 was determined using a Promega cytotoxicity kit, by measuring the released LDH in culture supernatants of (A) BCBL-1 and (B) HMVEC-d cells. (C) HMVEC-d cells serum-starved in the presence or absence of 1 μM C-646 for 2 h were washed and infected with KSHV for 2 h. DNA isolated from the nucleus of infected cells was evaluated for nuclear delivery of KSHV genome using real-time-DNA PCR. The nuclear viral DNA copy number was calculated using a standard curve generated from known concentrations of the ORF73 gene. (D, E and F) HMVEC-d cells serum-starved with or without 1 μM C-646 for 2 h were washed, infected with KSHV for 2 h, washed, and incubated with complete medium in the presence or absence of 1 μM C-646 for 24 h. (D) Cells were fixed, permeabilized, blocked with Image-iT FX signal enhancer, incubated with mouse anti-KSHV LANA-1 antibody and then probed with Alexa Fluor-488 conjugated secondary antibodies. White arrows indicate the LANA-1 dots in the nucleus of the infected cells and red arrows indicate uninfected cells. (E) The LANA-1 dots per infected cell were enumerated from at least 5 different fields with a minimum 10 cells and results plotted as a bar graph. (F and G) HMVEC-d cells serum-starved in the presence or absence of 1 μM C-646 for 2 h were either left uninfected or infected with KSHV (30 DNA copies/cell) for 2 h and incubated for 24 h in complete medium with or without 1 μM C-646. (F) Equal quantities of total cell lysate proteins in NETN buffer were western blotted with anti-acetylated antibody. (G) Equal quantities of whole cell lysates from the 24 h time point described above were IP-ed with anti-acetylated lysine antibody and western blotted for H2B. Total H2B and tubulin were used as input and loading controls, respectively. (TIF) [file ppat.1005019.s002.tif]

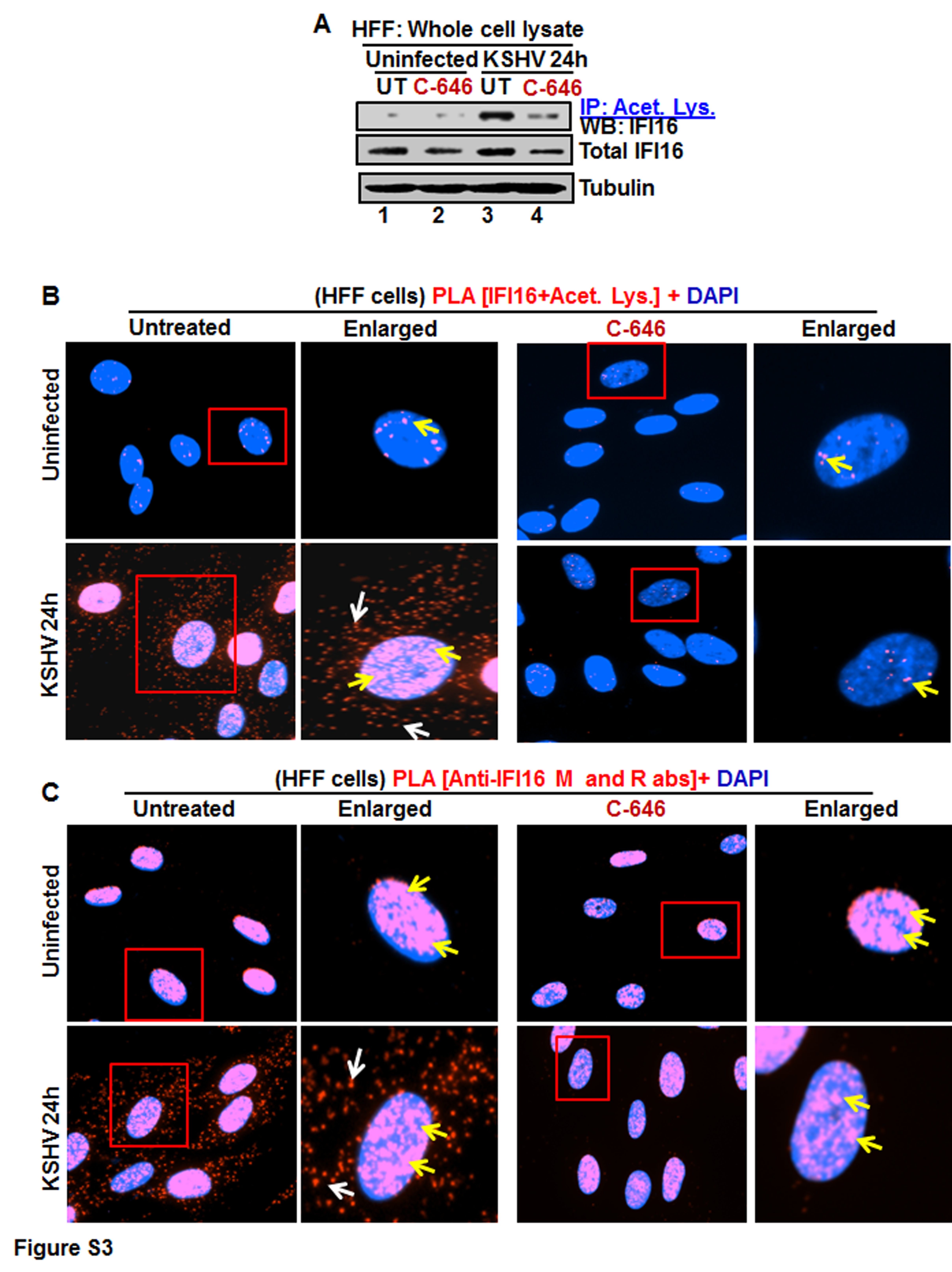

Supplement: S3 Fig — (A) HFF cells serum-starved in the presence or absence of 1 μM C-646 for 2 h were infected with KSHV (30 DNA copies/cell) for 2 h, washed, and incubated with complete medium for 24 h with or without 1 μM C-646. Equal amounts of total protein lysates in NETN-lysis buffer were IP-ed with anti-acetylated lysine antibodies and immunoblotted for IFI16. Total IFI16 and tubulin were used as loading controls. (B and C) HFF cells serum-starved in the absence or presence of 1 μM C-646 for 2 h were either left uninfected or infected with KSHV for 2 h, washed, cultured in complete medium for 24 h with or without 1 μM C-646 and subjected to PLA with anti-acetylated lysine and anti-IFI16 antibodies (B) or with anti-IFI16 mouse and rabbit antibodies (C). DAPI was used to stain the nucleus. Cytoplasmic and nuclear acetylated IFI16 in panel (B) denoted by white and yellow arrows, respectively. White and yellow arrows in panel (C) depict cytoplasmic and nuclear IFI16, respectively. (TIF) [file ppat.1005019.s003.tif]

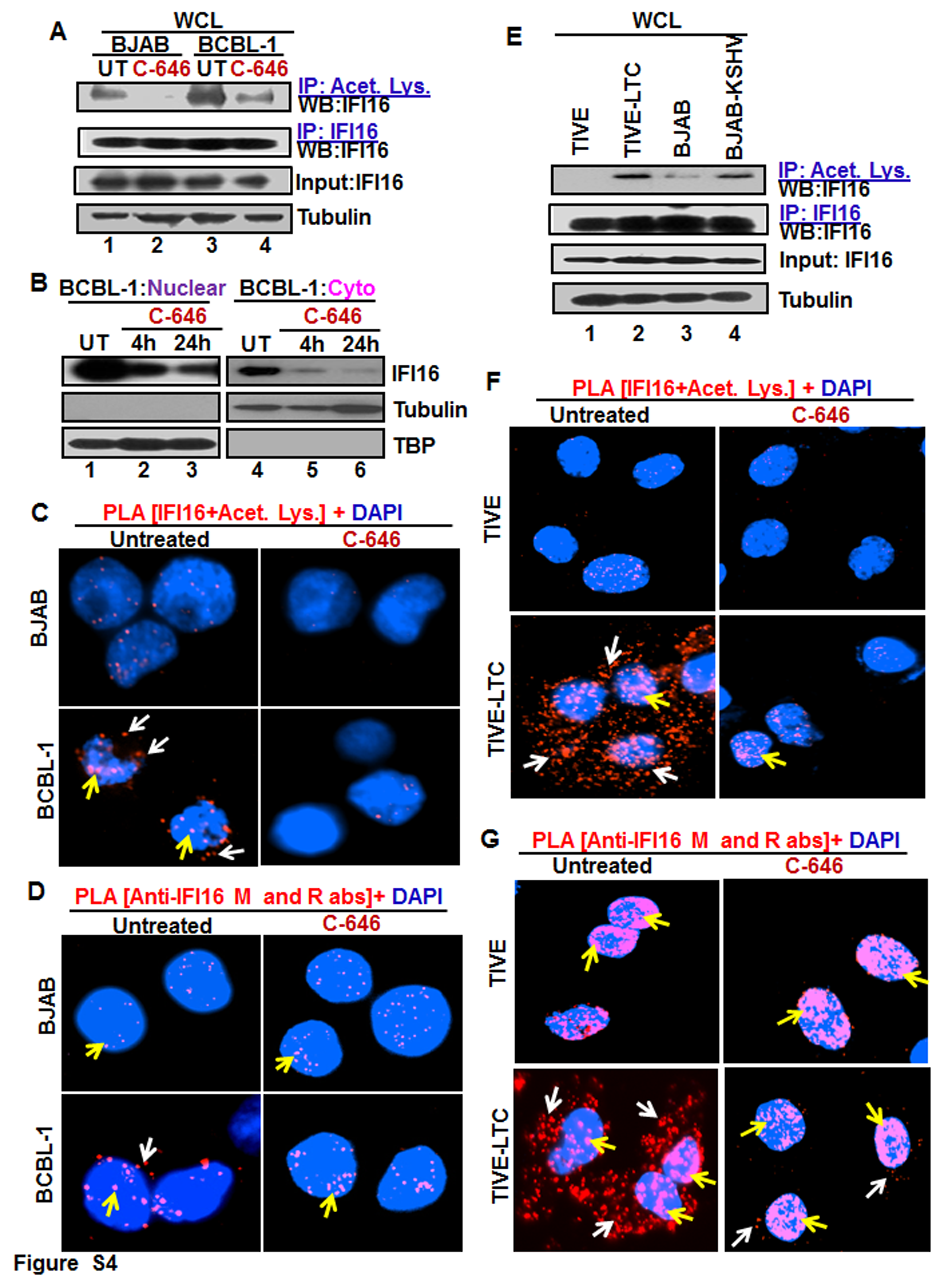

Supplement: S4 Fig — (A) BJAB (KSHV-) and BCBL-1 (KSHV+) cells were untreated or treated with 1 μM C-646 for 24 h, and WCL proteins in NETN buffer were IP-ed with anti-acetylated lysine antibodies and western blotted for IFI16. (B) The nuclear and cytoplasmic extracts from untreated BCBL-1 cells or cells treated with 1 μM C-646 for 4 and 24 h were western blotted for IFI16, TBP and tubulin. (C) BJAB and BCBL-1 cells in the presence or absence of 1 μM C-646 (24 h) were tested by PLA with anti-IFI16 and anti-acetylated lysine antibodies. White arrows and yellow arrows indicate cytoplasmic and nuclear acetylated IFI16, respectively. (D) BJAB and BCBL-1 cells left untreated or treated with 1 μM C-646 (24 h) were tested by PLA with anti-IFI16 mouse and rabbit antibodies. White and yellow arrows indicate cytoplasmic and nuclear IFI16, respectively. (E) WCL proteins in NETN buffer were IP-ed with anti-acetylated lysine antibodies and western blotted for IFI16. (F and G) TIVE and TIVE-LTC cells untreated or treated with 1 μM C-646 for 24 h were analyzed by PLA. (F) PLA using anti-IFI16 and anti-acetylated lysine antibodies. White arrows and yellow arrows indicate cytoplasmic and nuclear acetylated IFI16, respectively; (G) Anti-IFI16 mouse and rabbit antibodies. White arrows and yellow arrows indicate cytoplasmic and nuclear IFI16, respectively. (TIF) [file ppat.1005019.s004.tif]

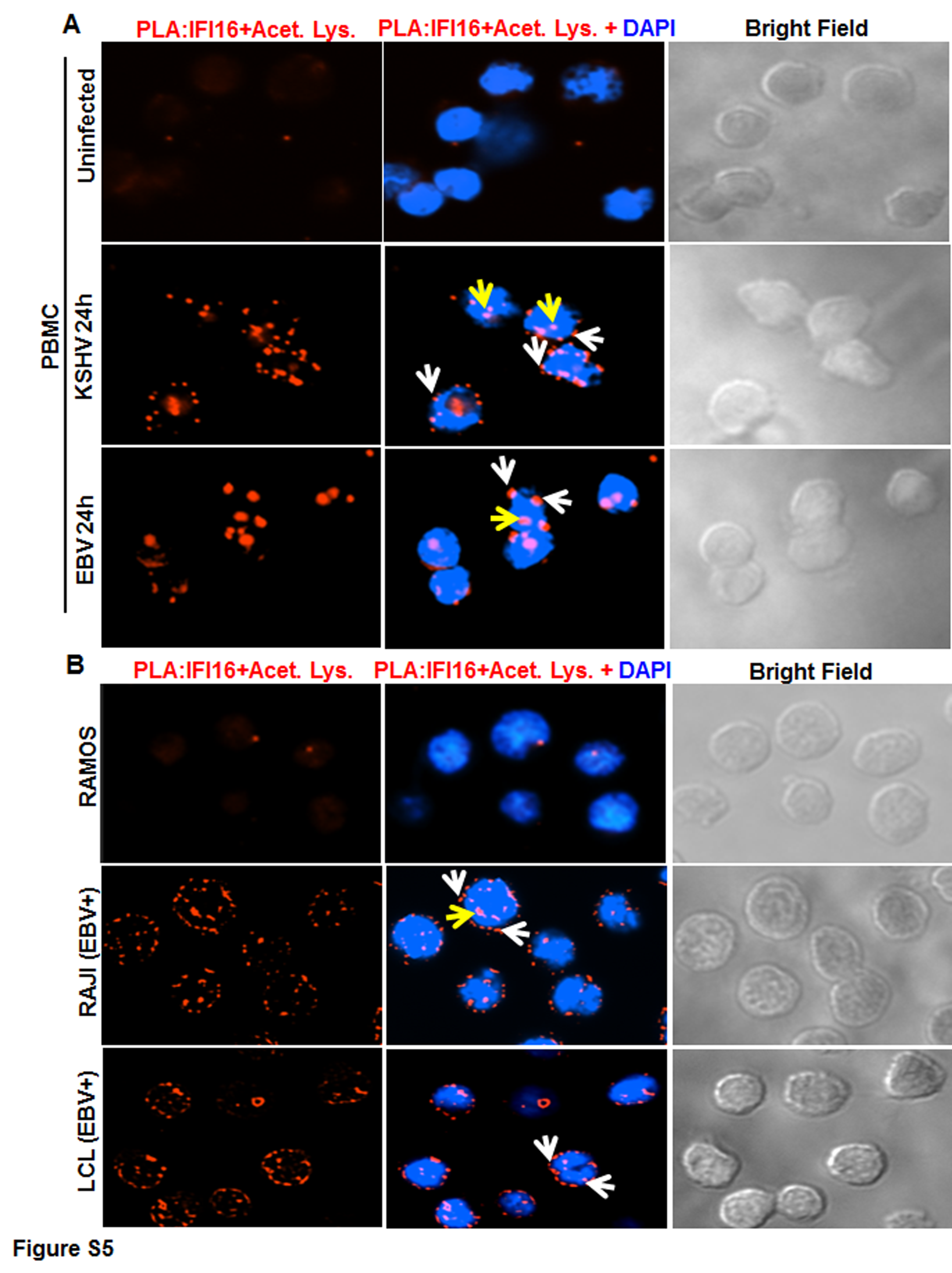

Supplement: S5 Fig — Human B cells (PBMCs) infected with KSHV or EBV for 24 h (A) and EBV negative Ramos and EBV positive RAJI and LCL cells (B) were fixed in acetone, blocked in Duolink blocking buffer, reacted with anti-IFI16 and anti-acetylated lysine antibodies and subjected to PLA. The acetylated IFI16 is represented as red dots. Yellow and white arrows depict nuclear and cytoplasmic acetylated IFI16, respectively. The right most panels show their corresponding bright field images. The images shown here are representative of three independent experiments. Magnification: 60X. (TIF) [file ppat.1005019.s005.tif]

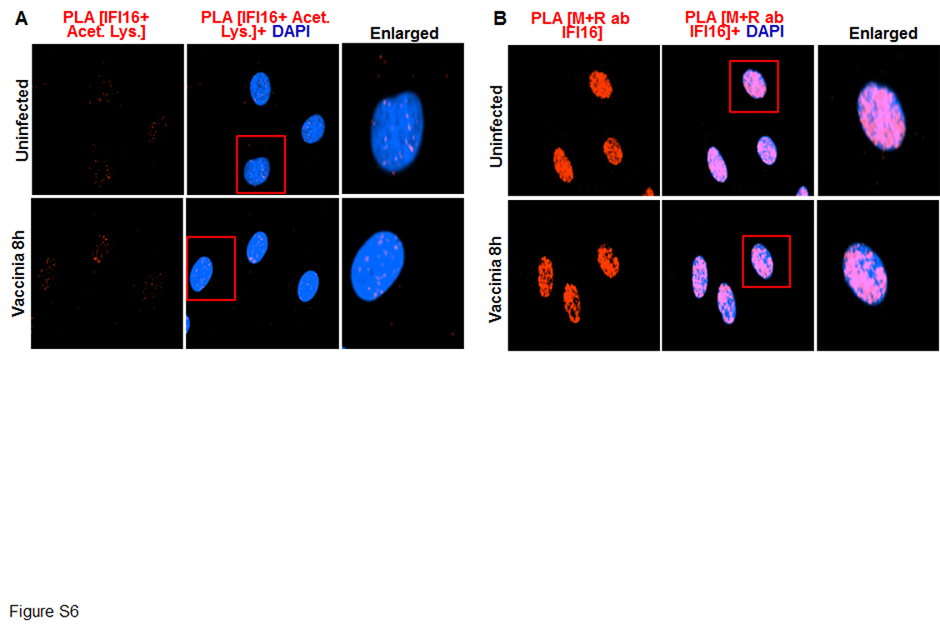

Supplement: S6 Fig — HMVEC-d cells were serum-starved in the absence or presence of 1 μM C-646 for 2 h, then either left uninfected or infected with Vaccinia virus (5 PFU/cell) for 2 h, washed, cultured in complete medium for 8 h with or without 1 μM C-646 and subjected to PLA with anti-acetylated lysine and anti-IFI16 antibodies (A) or with anti-IFI16 mouse and rabbit antibodies (B). DAPI was used to stain the nucleus. The red inset is enlarged in the right most panels. The images shown here are representatives of three independent experiments. Magnification: 60X. (TIF) [file ppat.1005019.s006.tif]

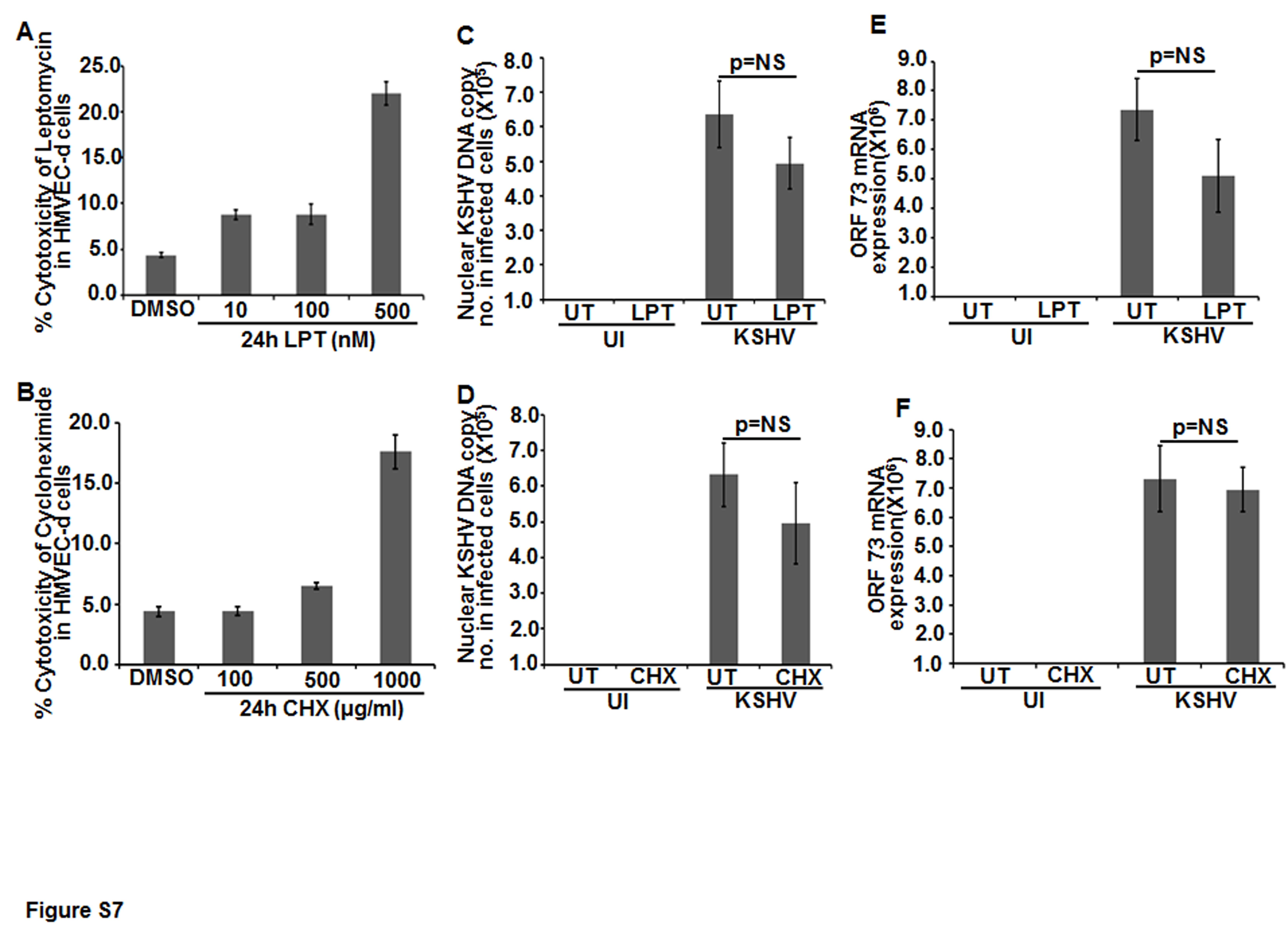

Supplement: S7 Fig — (A and B) The toxicity of various concentrations of Leptomycin B (LPT) and Cycloheximide (CHX) were evaluated in HMVEC-d cells. (C and D) HMVEC-d cells were serum- starved for 2 h in the presence or absence of 50 nM Leptomycin B or 200 μg/ml Cycloheximide, washed, left uninfected or infected with KSHV (30 DNA copies/cell) for 2 h and nuclei associated viral DNA copy numbers were estimated by real-time DNA PCR for the ORF73 gene. Results shown represent mean ± SD for three experiments. (E and F) HMVEC-d cells treated or untreated with 50 nM LPT or 200 μg/ml Cycloheximide during 2 h starvation were washed, infected with KSHV for 2 h, washed and incubated for 24 h in complete medium with or without inhibitors. RNA was subjected to qRT-PCR for ORF73 transcripts. Results shown represent mean ± SD for three experiments. (TIF) [file ppat.1005019.s007.tif]

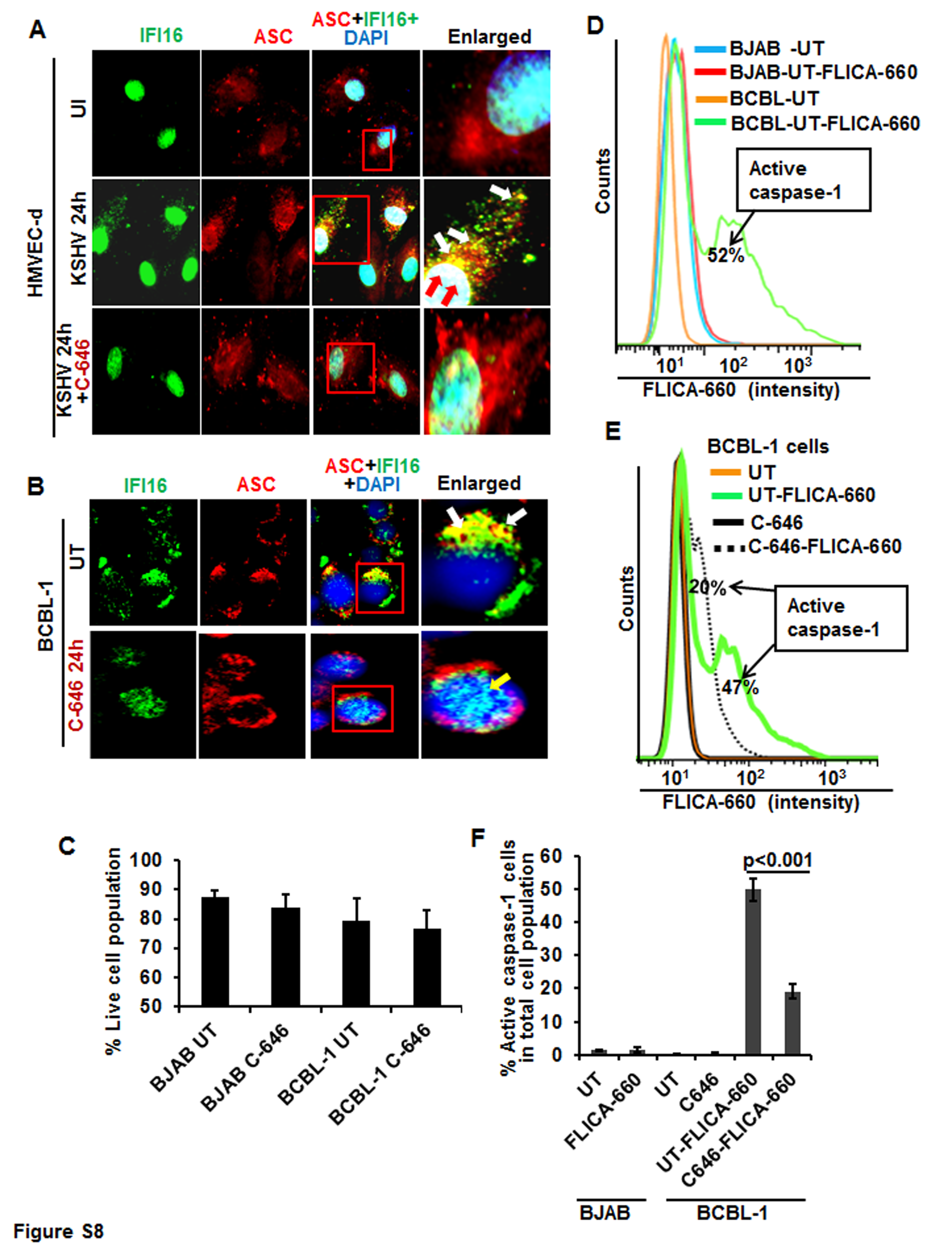

Supplement: S8 Fig — (A) HMVEC-d cells serum-starved with or without 1 μM C-646 were infected with KSHV for 2 h, washed, incubated in complete medium for 24 h in the presence or absence of 1 μM C-646 and IFA for IFI16 and ASC performed. DAPI was used to stain the nucleus. Insets in the merged panels are enlarged in the right most panels. The white and red arrows indicate the IFI16-ASC colocalization spots in the cytoplasm and nucleus, respectively. Magnification: 60X. (B) BCBL-1 cells untreated or treated with 1 μM C-646 were reacted with mouse anti-IFI16 and goat anti-ASC antibodies and then probed with Alexa Fluor-488 and Alexa Fluor-594 conjugated secondary antibodies, respectively. Nucleus was stained with DAPI. The white arrows depict the cytoplasmic IFI16-ASC colocalization. Images shown here are representative of three independent experiments. Magnification: 60X. (C) Percent live cells in BJAB and BCBL-1 cells in the absence or presence of 1 μM C-646 for 24 h were determined prior to FACS using the Trypan blue exclusion method. (D) The BJAB and BCBL-1 cells with active caspase-1 were enumerated using a FLICA 660-YVAD-FMK staining kit. Cells were washed in PBS, incubated with FLICA 660-YVAD-FMK dye, unbound dye removed by washing with PBS and analyzed by FACS. (E) BCBL-1 cells untreated or treated with 1 μM C-646 were examined for active caspase-1 using the above described kit and flow cytometry analysis was performed. (F) Percent active caspase-1 in total cell population of BJAB and BCBL-1 cells treated or untreated with 1 μM C-646 was enumerated using the data from FACS analysis and presented in the form of a bar graph. (TIF) [file ppat.1005019.s008.tif]

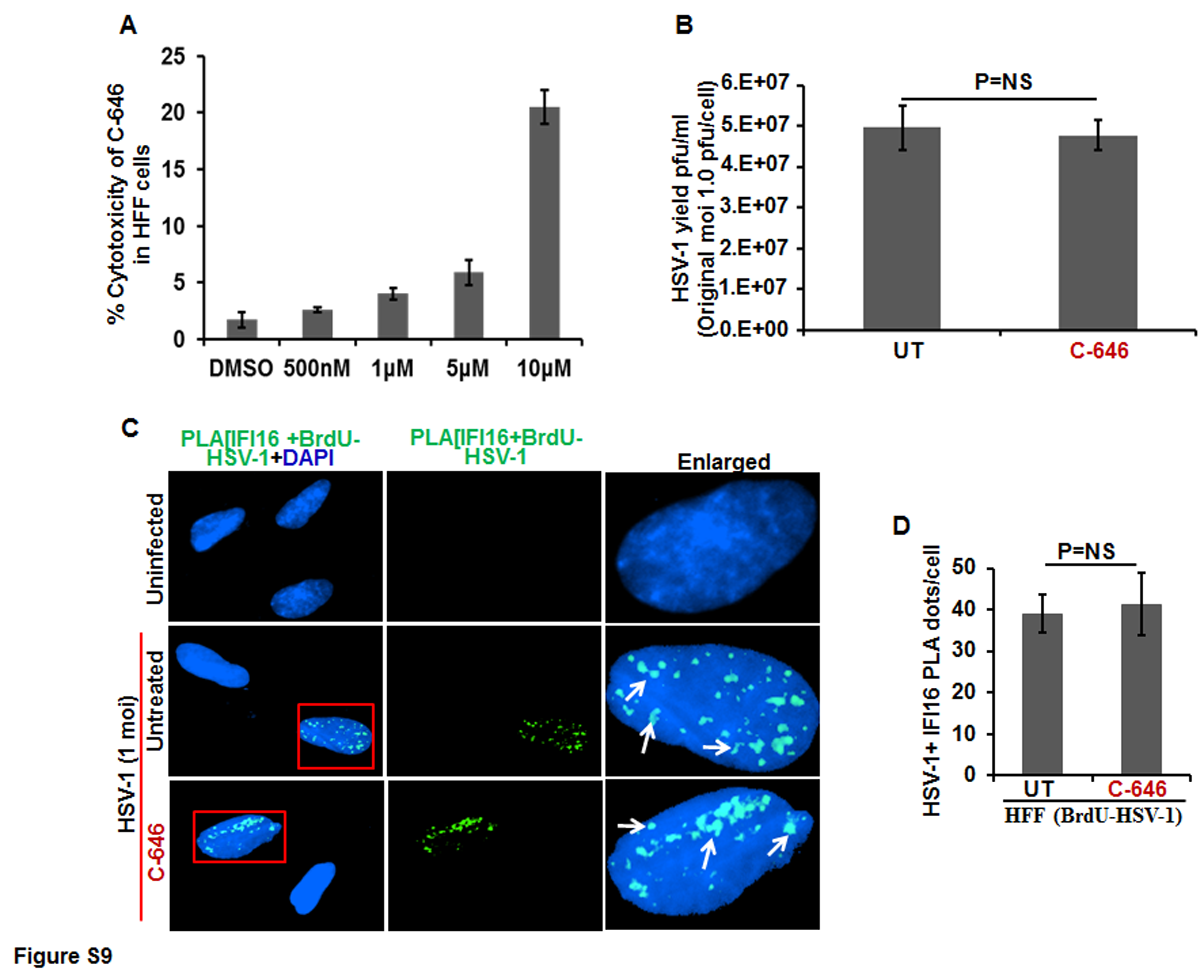

Supplement: S9 Fig — The cytotoxicity of various concentrations of C-646 was determined using a Promega cytotoxicity kit, by measuring the released LDH in culture supernatant of (A) HFF cells. (B) Vero cells serum-starved in the presence or absence of 1 μM C-646 for 2 h were washed and infected with HSV-1 (1 PFU/cell MOI) for 2 h then plaque assay was performed to assay the effect of C-646 on HSV-1 infectivity and production. (C) HFF cells pre-incubated with or without 1 μM C-646 for 2 h were washed, infected with BrdU genome labeled HSV-1 (1PFU/cell) for 2 h, washed and incubated with or without 1 μM C-646 for 4 h. Cells were subjected to PLA with anti-IFI16 and anti-BrdU antibodies. Boxed areas are enlarged. PLA spots indicated by the representative white arrows indicate the association between BrdU labeled HSV-1 genome and IFI16. The images shown here are representative of three independent experiments. Magnification: 60X. (D) PLA spots (C) from 10 fields with at least 3–4 cells/field were quantitated and presented here as a bar graph. (TIF) [file ppat.1005019.s009.tif]
